# Reconsidering repurposing: long-term metformin treatment impairs cognition in Alzheimer’s model mice

**DOI:** 10.1038/s41398-024-02755-9

**Published:** 2024-01-18

**Authors:** So Yeon Cho, Eun Woo Kim, Soo Jin Park, Benjamin U. Phillips, Jihyeon Jeong, Hyunjeong Kim, Christopher J. Heath, Daehwan Kim, Yurim Jang, Laura López-Cruz, Lisa M. Saksida, Timothy J. Bussey, Do Yup Lee, Eosu Kim

**Affiliations:** 1 Graduate School of Medical Science, Brain Korea 21 Project, Yonsei University College of Medicine, Seoul, 03722 Republic of Korea; 2 Department of Psychiatry, Laboratory for Alzheimer’s Molecular Psychiatry, Institute of Behavioral Science in Medicine, Yonsei University College of Medicine, Seoul, 03722 Republic of Korea; 3 Metabolism-Dementia Research Institute, Yonsei University College of Medicine, Seoul, 03722 Republic of Korea; 4 Graduate School of Medicine, Yonsei University, Seoul, 03722 Republic of Korea; 5 Department of Nursing, Seoyeong University, Gwangju, 61268 Republic of Korea; 6 Department of Agricultural Biotechnology, Seoul National University, Seoul, 08826 Republic of Korea; 7 Research Institute for Agricultural and Life Sciences, Seoul National University, Seoul, 08826 Republic of Korea; 8 Department of Psychology, The University of Cambridge, Cambridge, CB2 3EB UK; 9 School of Life, Health and Chemical Sciences, The Open University, Milton Keynes, MK7 6AA UK; 10 Interdisciplinary Program in Agricultural Genomics, Center for Food and Bioconvergence, Seoul National University, Seoul, 08826 Republic of Korea; 11 Robarts Research Institute, Schulich School of Medicine and Dentistry, Western University, London, N6A 5K8 Canada; 12 Department of Physiology and Pharmacology, Schulich School of Medicine and Dentistry, Western University, London, N6A 5C1 Canada

**Keywords:** Hippocampus, Long-term memory, Molecular neuroscience

## Abstract

Metformin, a primary anti-diabetic medication, has been anticipated to provide benefits for Alzheimer’s disease (AD), also known as “type 3 diabetes”. Nevertheless, some studies have demonstrated that metformin may trigger AD pathology and even elevate AD risk in humans. Despite this, limited research has elucidated the behavioral outcomes of metformin treatment, which would hold significant translational value. Thus, we aimed to perform thorough behavioral research on the prolonged administration of metformin to mice: We administered metformin (300 mg/kg/day) to transgenic 3xTg-AD and non-transgenic (NT) C57BL/6 mice over 1 and 2 years, respectively, and evaluated their behaviors across multiple domains via touchscreen operant chambers, including motivation, attention, memory, visual discrimination, and cognitive flexibility. We found metformin enhanced attention, inhibitory control, and associative learning in younger NT mice (≤16 months). However, chronic treatment led to impairments in memory retention and discrimination learning at older age. Furthermore, metformin caused learning and memory impairment and increased levels of AMPKα1-subunit, β-amyloid oligomers, plaques, phosphorylated tau, and GSK3β expression in AD mice. No changes in potential confounding factors on cognition, including levels of motivation, locomotion, appetite, body weight, blood glucose, and serum vitamin B12, were observed in metformin-treated AD mice. We also identified an enhanced amyloidogenic pathway in db/db mice, as well as in Neuro2a-APP_695_ cells and a decrease in synaptic markers, such as PSD-95 and synaptophysin in primary neurons, upon metformin treatment. Our findings collectively suggest that the repurposing of metformin should be carefully reconsidered when this drug is used for individuals with AD.

## Introduction

Alzheimer’s disease (AD) is typified by the progressive cognitive decline in multiple domains. As studies have revealed that patients with type 2 diabetes mellitus (DM) have an increased risk of AD, these two conditions are considered to share insulin resistance as a common pathogenic mechanism [1–5]. For this reason, anti-diabetic medication has been anticipated to provide benefits against the pathogenesis of AD, which is often referred to as “type 3 DM” [6, 7].

Metformin is a first-line anti-diabetic medication. As a potent insulin sensitizer, this drug draws various benefits on metabolism by activating AMP-activated protein kinase (AMPK), a pivotal enzyme for mitochondrial biogenesis and bioenergetic maintenance. Accordingly, metformin is among the most promising candidates for drug repurposing toward AD treatment [8]. However, several, if not all, studies have reported that metformin may trigger AD pathology in rodent models; metformin increases the expression of β-amyloid (Aβ) via beta-secretase 1 (BACE1) upregulation in C57BL/6 mice as well as in a transgenic AD model, 3xTg-AD mice [9] and promotes tau aggregation in mice with tauopathy [10]. Moreover, a study using primary neurons has shown that metformin leads to a reduction in dendritic spine density, recapitulating Aβ-induced synaptotoxicity [11] which is evoked via CaMKK2-dependent AMPK overactivation [12–14]. There have been epidemiological findings as well which support the relationship between metformin use and increased risk of AD [15, 16].

Considering the widespread usage of metformin globally [17], these prior findings indicate the imperative requirement for an in-depth scrutiny of this issue. However, most experimental studies have focused on the short-term effects of metformin, which may not accurately reflect the consequences of lifelong use in reality [18]. Furthermore, only a few studies have evaluated the behavioral and cognitive consequences of metformin treatment, often focusing on a single cognitive domain (usually memory) [10, 19]. As the diagnostic criteria for dementia require the severity of cognitive impairment as well as a minimum of two impaired cognitive domains [20], a neuropsychological battery with a multi-domain assessment is necessary for the diagnosis of dementia and the evaluation of its treatment outcomes [21, 22]. Above all, long-term behavioral outcomes would be translationally the most critical aspects of drug use, rather than short-term biological outcomes which may fluctuate within the homeostatic balance.

Therefore, we aimed to conduct a comprehensive behavioral study on long-term metformin treatment. We administered metformin to C57BL/6 (non-transgenic; NT) and transgenic 3xTg-AD mice throughout most of their lifespan and examined multi-domain cognitive functions using a touchscreen-based operant system. This system closely mirrors the human touchscreen neuropsychological test panel to enhance cross-species translational potential and utilize standardized protocols to ensure reproducibility [23–25]. Herein, we report that long-term metformin treatment causes impairments in multi-domain cognition in aged C57BL/6 as well as in AD mice.

## Materials and methods

### Animals

Male C57BL/6 mice were purchased from Orient Bio Inc. (Seongnam, Korea). Homozygous male and female 3xTg-AD (B6;129-Tg [APP^Swe^, tau^P301L^] 1Lfa Psen1^tm1Mpm^/Mmjax; MMRRC Stock No.34830-JAX; The Jackson Laboratory, Bar Harbor, ME, USA) mice were bred in our lab. All mice were housed in groups of 1–4 per cage in a specific pathogen-free room with a 12–h light/dark cycle (lights on from 8:00 a.m. to 8:00 p.m.) and humidity- and temperature-controlled environment. All procedures were approved by Yonsei University Health System Institutional Animal Care and Use Committee (IACUC) and performed in accordance with National Institute of Health guidelines for the Care and Use of Laboratory Animals. The experimental cohorts and the number of mice are described in Supplementary Materials and Methods and Supplementary Table 1, respectively.

### Drug administration

When the mice were 3 months old, 2 mg/mL metformin (1,1-dimethylbiguanide hydrochloride; Sigma Aldrich, St. Louis, MO, USA) was diluted in drinking water and orally administered. To avoid any reversive responses to metformin, the dose of metformin was gradually increased weekly. According to the daily water consumption of mice (~5 mL/mouse/day), the delivered dose of metformin was approximately 300 mg/kg/day, which could be converted to 2000 mg/person/day on a human clinical basis [9, 26]. The water and drug were changed every 3 or 4 days.

### Apparatus

All behavioral testing was conducted in standard Bussey–Saksida mouse touchscreen chambers (Campden Instruments Ltd., Loughborough, UK) as described elsewhere [24], and detailed descriptions are provided in Supplementary Materials and Methods.

### Shaping

The shaping procedure was conducted as described elsewhere [23, 24], and detailed descriptions were provided in Supplementary Materials and Methods.

### FR and PR schedule

The FR and PR schedule procedures were conducted as described elsewhere [27], and detailed descriptions are provided in Supplementary Materials and Methods. FR1, FR2, FR3, and FR5 sessions proceeded sequentially, and each session was completed within 60 min. FR5-uncapped (FR5-UC) was conducted after setting the baseline for 2 consecutive days. The mice performed the PR4 sessions for 3 consecutive days after the FR schedule. Each session of the PR schedule was terminated within 60 min or 5 min without any movement.

### 5-CSRT task

The 5-CSRT task procedure was conducted as described elsewhere [23, 28], and detailed descriptions are provided in Supplementary Materials and Methods. When all mice accomplished the criterion (completion of 60 trials within 60 min; 6-month-old C57BL/6 and 8-month-old 3xTg-AD mice: accuracy ≥80% and omission ≤20% for 2 consecutive days; 22-month-old C57BL/6 mice: accuracy ≥75% and omission ≤25% for 2 consecutive days), the baseline (SD = 2.0 s) was set for 2 consecutive days. The probe test was conducted for 4 consecutive days. To increase attentional demands, shorter SDs (2.0 s, 1.5 s, 1.0 s, and 0.5 s) were presented in a pseudo-random manner.

### PAL task

The PAL task procedure was conducted as described elsewhere [29], and detailed descriptions are provided in Supplementary Materials and Methods. When the group average accuracy of the dPAL task was higher than 80% (12-month-old C57BL/6 and 11-month-old 3xTg-AD mice) or 75% (22-month-old C57BL/6 mice) within 60 min, the sPAL task was proceeded for 3 consecutive days within 60 min. The sPAL retention sessions were conducted once a week for 3 weeks after the sPAL task.

### VD and reversal task

The VD and reversal task procedures were conducted as described elsewhere [30, 31], and detailed descriptions are provided in Supplementary Materials and Methods. When the mice achieved the criterion of the VD task (30 trials within 60 min; accuracy ≥ 80% for 2 consecutive days), the baseline was set for 2 consecutive days to proceed to the reversal task. When a mouse achieved the criterion of the reversal task (30 trials within 60 min; accuracy ≥80% for 2 consecutive days), it was subjected to the reversal retention session. Each mouse performed one retention session every 10 days after the last reversal session.

### Molecular works

Western blot analysis, immunohistochemistry, and metabolomic analysis were conducted as described in Supplementary Materials and Methods.

### Statistical analysis

Statistical analyses of behavioral and molecular data, except metabolomics data, were conducted using R version 3.6.3 and GraphPad Prism version 9 (Graphpad Software Inc., La Jolla, CA, USA). Comparison between the two groups’ differences was analyzed by *t* test. Repeated measured data were evaluated by repeated-measures (RM) analysis of variance analysis (ANOVA; when the number of groups was the same) or mixed effects model (when the number of groups was different). The survival rate was assessed by log-rank test. The correlation between the variables of the 5-CSRT task was calculated by Pearson’s correlation. All data were expressed as the mean ± SEM. Significance was set at α < 0.05.

## Results

### Metformin treatment enhances attention, inhibitory control, and associative learning in younger C57BL/6 mice

**Fig. 1:**
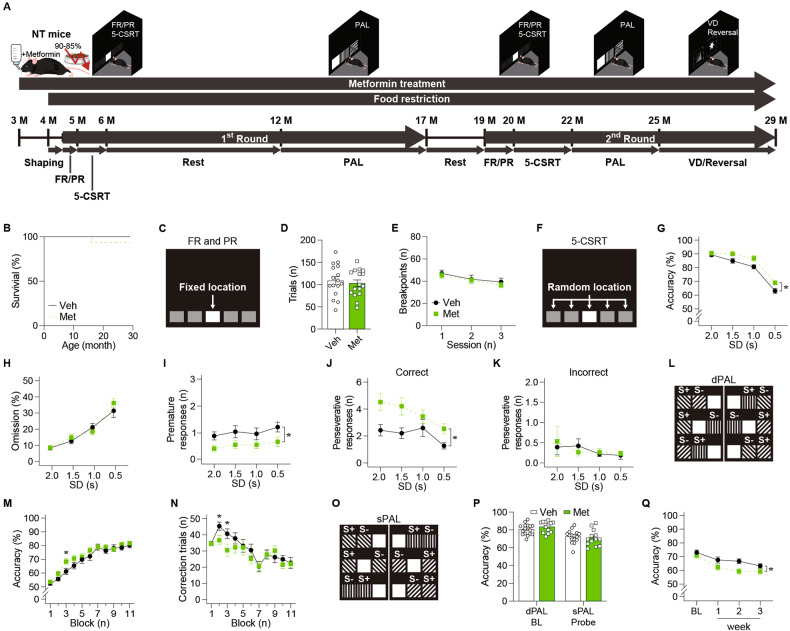
Metformin treatment enhanced cognition in younger NT mice and impaired cognition in older NT mice during the first round of behavioral assessment. **A** Behavioral experiment schedule of NT mice with metformin treatment. **B** The survival rate of NT mice (Veh: *n* = 16, Met: *n* = 16; *p* = 0.317; log-rank test) during the behavioral assessment. **C** Stimuli used in the FR and PR schedules. **D** The number of trials of 4-month-old NT mice (Veh: *n* = 16, Met: *n* = 16; *p* = 0.634; *t* test) in the FR schedule. **E** The breakpoint of 4-month-old NT mice (Veh: *n* = 16, Met: *n* = 16; *p* = 0.638; RM-ANOVA) in the PR schedule. **F** Stimuli used in the 5-CSRT task. **G** Accuracy (*p* = 0.022), (**H**) omission (*p* = 0.672), and the number of (**I**) premature responses (*p* = 0.026) and perseverative responses to (**J**) correct stimuli (*p* = 0.017) and (**K**) incorrect stimuli (*p* = 0.896; mixed effects model) of 6-month-old NT mice (Veh: *n* = 16, Met: *n* = 16) by SD in the 5-CSRT task. **L** Stimuli used in the dPAL task. **M** Accuracy (Main effect of group, *p* = 0.201; group by block interaction, *p* = 0.001; simple effect of group in block 3, *p* = 0.032) and (**N**) the number of correction trials (Main effect of group, *p* = 0.302; group by block interaction, *p* < 0.001; simple effect of group in block 2, *p* = 0.030; simple effect of group in block 3, *p* = 0.044; mixed effects model) of 12-month-old NT mice (Veh: *n* = 16, Met: *n* = 13) in the dPAL task. The block consisted of approximately 300 trials (288–324) as the number of trials per session gradually increased. **O** Stimuli used in the sPAL task. **P** Accuracy of 16-month-old NT mice (Veh, *n* = 16; Met, *n* = 13; dPAL, *p* = 0.209; sPAL, *p* = 0.517; *t* test) in the sPAL task. **Q** Accuracy of 16-month-old NT mice (Veh: *n* = 15, Met: *n* = 13; *p* = 0.032; mixed effects model) in the retention sessions of the sPAL task. Data are presented as mean ± SEM. *p < 0.05 versus Veh. NT non-transgenic C57BL/6 mice, FR fixed ratio, PR progressive ratio, 5-CSRT 5-choice serial reaction time, PAL paired associates learning, VD visual discrimination, Veh Vehicle, Met Metformin, SD stimulus duration, BL baseline.

As a previous study reported that metformin increases Aβ levels in C57BL/6 mice [9], we started behavioral testing in these mice with the same dosage used in that study (Fig. 1A). During the entire experimental period, there was no noticeable difference in life span between mice treated with metformin versus vehicle (Fig. 1B). First, we examined motivation levels for reward (strawberry milkshake [23]) since metformin can reduce appetite [32]. Total trial numbers in the fixed ratio (FR) and breakpoints in the progressive ratio (PR) schedules indicated no significant group difference in motivation levels (Fig. 1C–E). No group differences were observed in blank touch numbers (reflective of non-specific behavioral activation) and in locomotor activity during the FR/PR testing (Supplementary Fig. 1). These results indicate that motivation and motor function that are needed to engage mice in behavioral tasks were not altered by metformin, justifying further behavior experiments.

We next tested attention using the 5-choice serial reaction time (5-CSRT) task (Fig. 1F). In pretraining, groups did not differ in the number of sessions required to reach the criterion (to enter the probe test), showing comparable task rule learning (Supplementary Fig. 2A). In the probe test where shorter stimulus durations (SDs) were implemented to tax attentional load, metformin-treated mice exhibited higher response accuracy than vehicle-treated mice, with a comparable omission rate (Fig. 1G, H, and Supplementary Fig. 2B, C). Group differences in accuracy became apparent in shorter (more difficult) SD conditions than the baseline SD (2.0 s; Fig. 1G), supporting that 5-CSRT task outcomes may reflect the genuine difference in attention rather than the difference resulting from other confounding factors. Premature responses (indicating impulsivity) were lower in metformin-treated mice, indicating improved inhibitory control (Fig. 1I, Supplementary Fig. 2D). Perseverative (repetitive) responses to correct, but not to incorrect, stimuli were also higher in metformin-treated mice (Fig. 1J, K, Supplementary Fig. 2E, F). However, metformin did not affect locomotor behaviors indexed by beam break rates and response time (latencies) (Supplementary Fig. 2G–J). So, we explored the relationship between performance scores of 5-CSRT subdomains by generating heatmaps (Supplementary Fig. 2K, L). We found that the perseveration rate to correct stimuli was positively associated with accuracy scores in metformin-treated mice but not in vehicle-treated mice. Thus, it is unlikely that the increased number of repetitive responses to correct stimuli reflected inappropriate response perseveration in metformin-treated mice. Taken together, our data suggest that metformin enhanced frontal lobe-linked executive functions in young NT mice.

We then evaluated temporal lobe-dependent learning and memory using the object-location paired-associates learning (PAL) task with different pairs of objects (dPAL; Fig. 1L). We found that metformin facilitated learning performance, indicated by significant drug (group) by time (block) interactions on accuracy and correction trials (Fig. 1M, N). When the PAL performance of all mice reached ≥ 80% accuracy, we conducted the same PAL (sPAL) task with the same object pairs to rule out mediation effect (e.g., “if A and B are presented together, choose the left side”; Fig. 1O). Performance on sPAL of each group was not significantly lower than dPAL performance, indicating that mice performed depending on object-location associations. Performance in sPAL were comparable between groups (Fig. 1P). Finally, we conducted the retention sessions with the stimuli used in sPAL to test memory retention in NT mice at 17 months of age. Interestingly, we found that memory retention was poorer in metformin-treated mice (Fig. 1Q). Although locomotor functions were not generally affected during the whole PAL task performance, response latency was significantly lower in metformin-treated mice, indicating an enhanced willingness to respond (Supplementary Fig. 3). Together, these findings indicate that metformin facilitated learning speed in middle age (12–14 months of age) without affecting maximum performance levels of learning, but impaired retention of already learned information in longer-treated, older NT mice (17 months of age).

### Chronic metformin treatment impairs visual discrimination and memory retention in older C57BL/6 mice

**Fig. 2:**
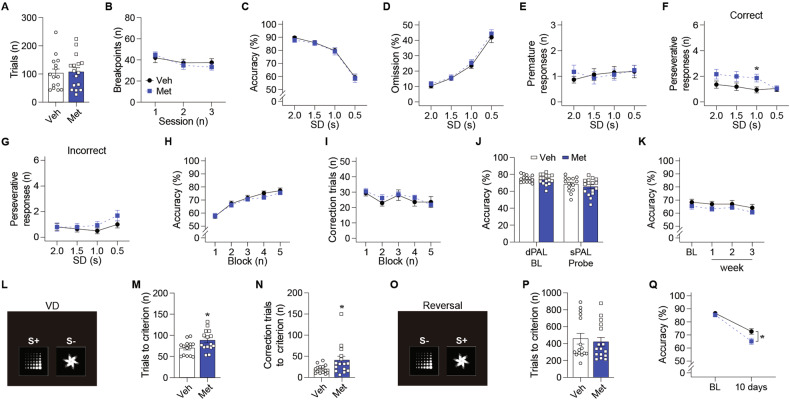
Prolonged metformin treatment impaired cognition in older NT mice during the second round of behavioral assessment. **A** The number of trials of 20-month-old NT mice (Veh: *n* = 15, Met: *n* = 16; *p* = 0.825; *t* test) in the FR schedule. **B** The breakpoint of 20-month-old NT mice (Veh: *n* = 15, Met: *n* = 16; *p* = 0.761; RM ANOVA) in the PR schedule. **C** Accuracy (*p* = 0.721), (**D**) omission (*p* = 0.522), and the number of (**E**) premature responses (*p* = 0.889) and perseverative responses to (**F**) correct stimuli (Main effect of group, *p* = 0.076; group by SD interaction, *p* = 0.046; simple effect of group in SD 1.0 s, *p* = 0.023) and (**G**) incorrect stimuli (*p* = 0.286; mixed effects model) of 22-month-old NT mice (Veh: *n* = 15, Met: *n* = 16) by SD in the 5-CSRT task. **H** Accuracy (*p* = 0.623) and (**I**) the number of correction trials (*p* = 0.637; mixed effects model) of 22-month-old NT mice (Veh: *n* = 14, Met: *n* = 16) in the dPAL task. The block consisted of approximately 300 trials (288–324) as the number of trials per session gradually increased. **J** Accuracy of 24-month-old NT mice (Veh, *n* = 14; Met, *n* = 16; dPAL, *p* = 0.825; sPAL, *p* = 0.336; *t* test) in the sPAL task. **K** Accuracy of 25-month-old NT mice (Veh: *n* = 14, Met: *n* = 16; *p* = 0.219; mixed effects model) in the retention sessions of the sPAL task. **L** Stimuli used in the VD task. The number of (**M**) trials (*p* = 0.025) and (**N**) correction trials (*p* = 0.046; *t* test) to accomplish the criterion of 25-month-old NT mice (Veh: *n* = 14, Met: *n* = 15) in the VD task. **O** Stimuli used in the reversal task. **P** The number of trials to accomplish the criterion of 26-month-old NT mice (Veh: *n* = 15, Met: *n* = 16; *p* = 0.650; *t* test) in the reversal task. **Q** Accuracy of 29-month-old NT mice (Veh: *n* = 15, Met: *n* = 16; *p* = 0.025; mixed effects model) in the reversal retention session. Data are presented as mean ± SEM. *p < 0.05 versus Veh. NT, non-transgenic C57BL/6 mice; Veh Vehicle, Met Metformin, SD stimulus duration, BL baseline, VD visual discrimination.

As we observed retention memory impairment in older mice with metformin, we conducted another (the second) round of behavioral assessments starting at 19 months of age to investigate the effects of metformin in older age (Note that the first and second rounds were conducted in the same cohort; Fig. 1A). Mice exhibited no difference in FR and PR performances (Fig. 2A, B and Supplementary Fig. 4). The second round of the 5-CSRT task in older age revealed no significant group differences in any task measures, except higher perseveration in metformin-treated mice (Fig. 2C–G and Supplementary Fig. 5), which was consistent with the first-round results (Fig. 1J). Unlike in the first round (Fig. 1M–Q), metformin did not affect learning speed or memory retention in the second round of the PAL task (Fig. 2H–K and Supplementary Fig. 6A–F). Lastly, we conducted the visual discrimination (VD) and reversal learning tasks (Fig. 2L, O). Metformin-treated mice (25 months of age) exhibited an impairment in VD learning (Fig. 2M, N and Supplementary Fig. 6G–I). However, performance in reversal learning was comparable between groups (Fig. 2P and Supplementary Fig. 6J–M), indicating no difference in cognitive flexibility. However, when we conducted the retention test 10 days after the baseline sessions, metformin-treated mice (29 months old) showed impairment in retention memory (Fig. 2Q and Supplementary Fig. 6N, O). Together, these findings indicate that chronic metformin treatment impairs discrimination learning and memory retention in older NT mice.

### Chronic metformin treatment impairs associative learning in 3xTg-AD mice

**Fig. 3:**
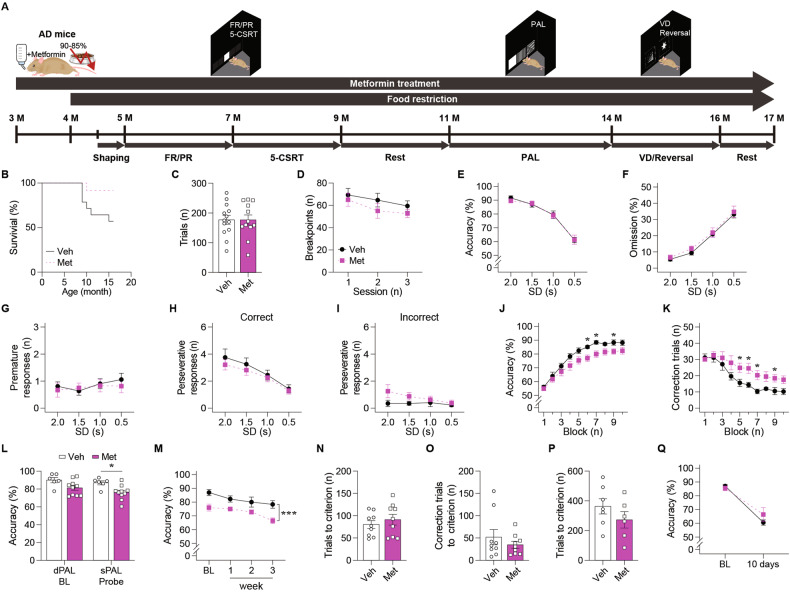
Long-term metformin treatment exacerbated the cognitive decline in AD mice. **A** Behavioral experiment schedule of AD mice with metformin treatment. **B** The survival rate of AD mice (Veh: *n* = 14, Met: *n* = 12; *p* = 0.052; log-rank test) during the behavioral assessment. **C** The number of trials of 5-month-old AD mice (Veh: *n* = 13, Met: *n* = 12; *p* = 0.988; *t* test) in the FR schedule. **D** The breakpoint of 6-month-old AD (Veh: *n* = 13, Met: *n* = 12; *p* = 0.346; RM ANOVA) in the PR schedule. **E** Accuracy (*p* = 0.838), (**F**) omission (*p* = 0.514), and the number of (**G**) premature responses (*p* = 0.732) and perseverative responses to (**H**) correct stimuli (*p* = 0.488) and (**I**) incorrect stimuli (*p* = 0.090; mixed effects model) of 9-month-old AD mice (Veh: *n* = 13, Met: *n* = 12) by SD in the 5-CSRT task. **J** Accuracy (Main effect of group, *p* = 0.054; main effect of block, *p* < 0.001; group by block interaction, *p* = 0.017; simple effect of group in block 6, *p* = 0.021; simple effect of group in block 7, *p* = 0.019; simple effect of group in block 9, *p* = 0.043) and (**K**) the number of correction trials (Main effect of group, *p* = 0.104; main effect of block, *p* < 0.001; group by block interaction, *p* < 0.001; simple effect of group in block 5, *p* = 0.040; simple effect of grou*p* in block 6, *p* = 0.029; simple effect of grou*p* in block 7, *p* = 0.020; simple effect of grou*p* in block 9, *p* = 0.036; mixed effects model) of 13-month-old AD mice (Veh: *n* = 6, Met: *n* = 10) in the dPAL task. The block consisted of approximately 300 trials (288–324) as the number of trials per session gradually increased. **L** Accuracy of 13-month-old AD mice (Veh, *n* = 6; Met, *n* = 10; dPAL, *p* = 0.053; sPAL, *p* = 0.011; *t* test) in the sPAL task. **M** Accuracy of 14-month-old AD mice (Veh, *n* = 6; Met, *n* = 10; *p* < 0.001; mixed effects model) in the retention sessions of sPAL task. The number of (**N**) trials (*p* = 0.480) and (**O**) correction trials (*p* = 0.379; *t* test) to accomplish the criterion of 14-month-old AD mice (Veh: *n* = 9, Met: *n* = 9) in the VD task. **P** The number of trials to accomplish the criterion of 14-month-old AD mice (Veh: *n* = 7, Met: *n* = 6; *p* = 0.256; *t* test) in the reversal task. **Q** Accuracy of 16-month-old AD mice (Veh: *n* = 7, Met: *n* = 6; *p* = 0.499; mixed effects model) in the reversal retention session. Data are presented as mean ± SEM. *p < 0.05, **p < 0.01, ***p < 0.001 versus Veh. AD, 3xTg-AD mice, FR fixed ratio, PR progressive ratio, 5-CSRT 5-choice serial reaction time, PAL paired associates learning, VD visual discrimination, Veh Vehicle, Met Metformin, SD stimulus duration, BL baseline.

We now assessed cognition in 3xTg-AD mice (Fig. 3A). During the entire treatment period, metformin-treated mice showed a trend towards a longer life span (Fig. 3B). The performance in FR and PR schedules indicated no group differences in motivation and locomotor functions (Fig. 3C, D, and Supplementary Fig. 7). Next, we conducted the 5-CSRT task. In pretraining, the number of sessions to reach the performance criterion did not differ between groups (Supplementary Fig. 8A). In the probe test, behavioral performances were also comparable between groups, suggesting no significant effects of metformin on frontal/executive functions in AD mice (Fig. 3E–I and Supplementary Fig. 8B–K). However, the PAL task revealed that metformin slowed paired-associates learning in AD mice, as indicated by a significant interaction between group (drug) and time (block) in terms of accuracy and correction trials (Fig. 3J, K). After AD mice were trained until both groups reached comparable levels of accuracy in dPAL, the probe sPAL was conducted, revealing a significant group difference (Fig. 3L). Subsequent retention tests also indicated that impaired performance of PAL persisted in metformin-treated AD mice (Fig. 3M). Although response latency was significantly increased by metformin during the sPAL retention sessions, groups did not overall differ in locomotor functions (Supplementary Fig. 9A–F). Metformin treatment affected neither VD nor reversal learning performances (Fig. 3N–P and Supplementary Fig. 9G–M). Finally, the retention test with the reversal task stimuli exhibited no between-group difference (Fig. 3Q and Supplementary Fig. 9N, O). In sum, these findings indicate that chronic metformin treatment causes associative learning impairments in AD mice.

### Chronic metformin treatment upregulates hippocampal AMPKα1-subunit in 3xTg-AD mice

**Fig. 4:**
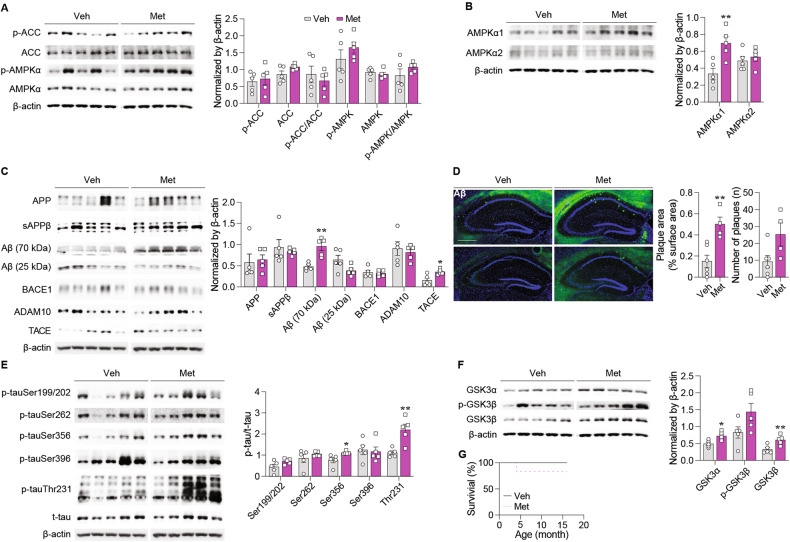
Long-term metformin treatment upregulated AMPKα1 expression and aggravated AD pathology in AD mice. Western blot analysis band images and quantification of (**A**) ACC and AMPKα expression and their phosphorylation levels (p-ACC, *p* = 0.709; ACC, *p* = 0.589; p-ACC/ACC, *p* = 0.525; p-AMPKα, *p* = 0.318; AMPKα, *p* = 0.535; p-AMPKα/AMPKα, *p* = 0.280), (**B**) AMPKα1- and α2-subunit expression (AMPKα1, *p* = 0.008; AMPKα2, *p* = 0.550), and (C) APP, sAPPβ, Aβ, ADAM10, TACE, and BACE1 expression [APP, *p* = 0.781; sAPPβ, *p* = 0.622; Aβ (70 kDa), *p* = 0.002; Aβ (25 kDa), *p* = 0.074; ADAM10, *p* = 0.626; TACE, *p* = 0.014; BACE1, *p* = 0.742; *t* test] in the hippocampus of 17-month-old AD mice (Veh: *n* = 5, Met: *n* = 5). **D** Representative immunohistochemistry images of D56D2-positive Aβ deposition and quantification of the percentage of Aβ plaque-positive area and the number of Aβ plaques in the hippocampus of 17-month-old AD mice (Veh: *n* = 6, Met: *n* = 4; area, *p* = 0.005; number; *p* = 0.052; *t* test). Scale bar: 500 μm. Western blot analysis band images and quantification of (**E**) p-tau and t-tau expression (**F**) GSK3α, GSK3β, and phosphorylated GSK3β expression (GSK3α, *p* = 0.015; p-GSK3β, *p* = 0.081; GSK3β, *p* = 0.009; *t* test) in the hippocampus of 17-month-old AD mice (Veh: *n* = 5, Met: *n* = 5). **G** The survival rate of AD mice (Veh: *n* = 6, Met: *n* = 6; *p* = 0.317; log-rank test) during metformin treatment. Data are presented as mean ± SEM. *p < 0.05, **p < 0.01 versus Veh. Veh, Vehicle, Met Metformin.

Calorie restriction is known to activate AMPK [33]. Thus, to exclude the confounding effects of food restriction on AMPK activity, all molecular investigations were conducted with free-fed female AD mice that were littermates of male AD mice that participated in behavioral experiments. As disrupted AMPK function and altered AMPK subunit expression have been associated with AD pathology [34, 35], we examined the levels of AMPK, phospho-AMPK (p-AMPK; active form), ACC (substrate of AMPK), p-ACC, and AMPKα subunit expression in the hippocampal tissue. We found no significant group differences in AMPK activity levels (Fig. 4A), but, interestingly, a significant increase in AMPKα1 expression upon metformin treatment (Fig. 4B). As the latter finding has been reported in human AD brain [36], our findings suggest that metformin-induced cognitive impairment might be associated with increased expression of hippocampal AMPKα1-subunit in AD mice.

### Chronic metformin treatment exacerbates AD pathology in 3xTg-AD mice

We examined the hallmarks of AD pathologies. We found that chronic metformin treatment significantly increased the levels of Aβ oligomers (70 kDa) (Fig. 4C) as well as Aβ plaques (Fig. 4D). AMPK may act as a tau kinase [36]. So, metformin significantly increased the levels of p-tau at Ser356 and Thr231 (Fig. 4E). Glycogen synthase kinase 3β (GSK3β), a major tau kinase [37, 38], can be phosphorylated (inhibited) by AMPK [39, 40]. Also, GSK3α has been shown to be involved in AD pathology [41–43]. The levels of GSK3α and GSK3β, but not of p-GSK3β, were significantly increased by metformin treatment (Fig. 4F). Life span of free-fed AD mice was not altered by metformin treatment (Fig. 4G). These findings suggest that chronic metformin treatment exacerbates Aβ and tau pathologies in AD mice.

### Metformin treatment triggers AD-associated pathology in vitro

As increased levels of Aβ oligomer and plaque were observed in vivo molecular analysis, we examined changes in the expression of secretases involved in the amyloidogenic pathway using cells. In Neuro2a, metformin upregulated BACE1 and downregulated TACE expression, paralleled with increased AMPK activity in a dose-dependent manner (Supplementary Fig. 10A, B). Consistently, sAPPβ and secreted Aβ levels were increased in the Neuro2a-APP_695_ stable cell line (Supplementary Fig. 10C). Since metformin is originally an anti-diabetic drug, we additionally treated metformin to db/db (type 2 diabetes model) mice and found enhanced amyloidogenic pathway (Supplementary Fig. 11).

Next, we explored changes in synaptic markers and morphology using primary neuronal culture to see if these are consistent with our in vivo behavioral findings. We found that 72h-treatment of metformin significantly decreased PSD-95, synaptophysin, NeuN, and MAP2 in a dose-dependent manner at DIV 18 (Supplementary Fig. 12). These findings suggest that metformin-induced cognitive impairment might be associated with synaptic toxicity, as well as with upregulated amyloidogenic pathway.

### Potential causes of cognitive impairment ruled out

We measured the concentration of metformin in drinking water and mouse serum to validate whether metformin had been administered adequately. Metformin concentration was maintained stably in the drinking water for 3 or 4 days (473.97 ± 32.120 μM and 428.01 ± 76.536 μM, respectively) compared to that on day 0 (633.95 ± 147.575 μM; *n* = 3). Metformin was also found in mouse bodies since the serum concentration of metformin was 15.43 ± 2.549 μM and 9.60 ± 3.239 μM in NT and AD mice, respectively (*n* = 4).

**Fig. 5:**
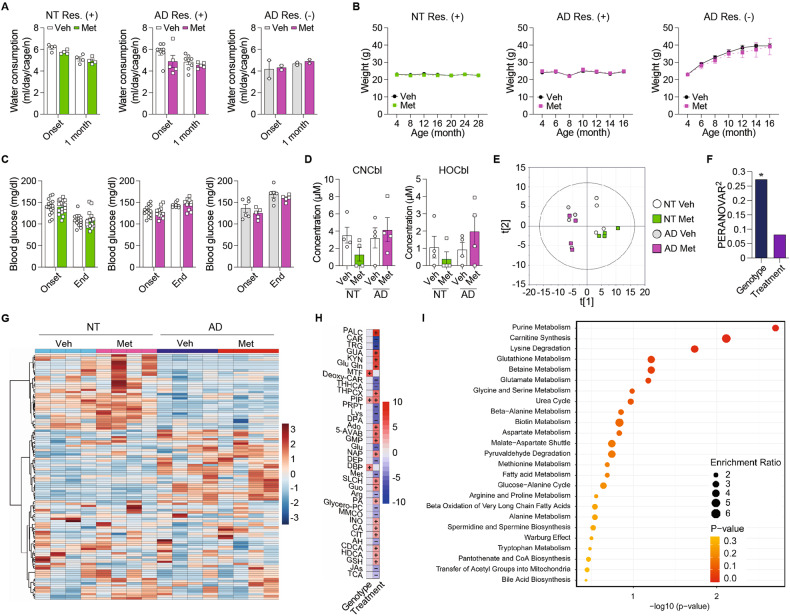
Long-term metformin treatment caused no adverse outcomes in NT and AD mice. **A** Water consumption of NT mice with food restriction (Veh: *n* = 4, Met: *n* = 4; *p* = 0.170; RM-ANOVA), AD mice with food restriction (Veh: *n* = 8, Met: *n* = 5; *p* = 0.160; mixed effects model), and AD mice without food restriction (Veh: *n* = 2, Met: *n* = 2; *p* = 0.696; RM-ANOVA). **B** Weight of NT mice with food restriction (Veh: *n* = 16, Met: *n* = 16; *p* = 0.634; mixed effects model), AD mice with food restriction (Veh: *n* = 14, Met: *n* = 12; *p* = 0.792; mixed effects model), and AD mice without food restriction (Veh: *n* = 6, Met: *n* = 5; *p* = 0.589; RM-ANOVA). **C** Fasting blood glucose concentration of NT mice with food restriction (Veh: *n* = 16, MET: *n* = 16; *p* = 0.996; mixed effects model), AD mice with food restriction (Veh: *n* = 14, Met: *n* = 12; *p* = 0.849; mixed effects model), and AD mice without food restriction (Veh: *n* = 6, Met: *n* = 5; *p* = 0.178; RM-ANOVA). **D** CNCbl and HOCbl concentration in the serum of NT and AD mice (*n* = 4 per group; CNCbl, NT mice, *p* = 0.113; AD mice, *p* = 0.615; HOCbl, NT mice, *p* = 0.397; AD mice, *p* = 0.321; *t* test). **E** Score scatter plot showing the distribution of vehicle- and metformin-treated NT and AD mice based on principal component analysis. **F** Permutational multivariate analysis of variance showing changes in serum metabolites of vehicle- and metformin-treated NT and AD mice clustered by group and genotype (genotype, r^2^ = 0.272, *p* < 0.001; treatment, r^2^ = 0.08, *p* = 0.204). **G** Heatmap of hierarchical clustering analysis showing chemically and biochemically classified metabolites of serum from vehicle- and metformin-treated NT and AD mice clustered by group and genotype. **H** Multiple linear regression analysis showing changes in serum metabolites of vehicle- and metformin-treated NT and AD mice. Full names of metabolites were shown in Table S2. **I** Metabolite set enrichment analysis showing changes in serum metabolites of vehicle- and metformin-treated groups of NT mice. Data are presented as mean ± SEM. *p < 0.05, **p < 0.01 versus Veh. Veh, vehicle; Met metformin, NT non-transgenic C57BL/6 mice, AD 3xTg-AD mice, Res food restriction, CNCbl cyanocobalamin, HOCbl, hydroxocobalamin.

Next, we explored potential adverse effects of metformin as confounding factors on behavioral outcomes. First, we examined appetite or digestion [32] by measuring water consumption (Fig. 5A) and body weight (Fig. 5B), which did not differ between groups. Further, blood glucose levels did not differ (Fig. 5C), ruling out hypoglycemia-induced cognitive impairment [44]. Vitamin B12 deficiency is another serious adverse effect of long-term metformin use [45]. This vitamin is crucially involved in normal brain function through one-carbon metabolism [46]. Thus, we conducted not only targeted (cobalamins) but also untargeted metabolic profiling in the serum to explore changes in metabolism thoroughly. Among the different forms of vitamin B12, cyanocobalamin and hydroxocobalamin were within the range of detection, and their levels in the serum did not significantly differ between groups (Fig. 5D). Untargeted metabolite profiles analyzed by principal component analysis (PCA) showed that the metabolic phenotype was clearly separated by genotype (NT vs. AD mice) but not by treatment (vehicle vs. metformin) (Fig. 5E). Permutational multivariate analysis of variance (PERMANOVA) was applied to quantify the explained variances of serum metabolome by genotype and treatment. Genotype explained that 27% of the metabolomic variance was significantly associated, but the treatment did not (Fig. 5F). Further, hierarchical clustering analysis (HCA) consistently showed distinct profiles between NT and AD mice (Fig. 5G). Next, we sought metabolites with genotype- or treatment-specific changes based on a multiple linear regression model. A total of 35 metabolites were significantly associated with genotype after Benjamini–Hochberg correction (*q* < 0.05). On the contrary, only three compounds (metformin, L-pipecolic acid, and dibutyl phthalate) showed a significant association with treatment (Fig. 5H). The enrichment analysis of the genotype-associated metabolites showed significant enrichment of purine metabolism, carnitine synthesis, and lysine degradation (Fig. 5I), whereas no enriched pathway was determined for treatment-associated metabolites. Together, these findings argue against metformin-induced alterations in systemic metabolism as a primary cause of cognitive impairment.

## Discussion

We sought to examine the long-term effects of metformin treatment on multi-domain cognition in AD mice. Previous studies examining the potential of repurposing metformin toward AD treatment have provided highly conflicting results [47–49], some of which have alerted that metformin can even trigger or aggravate AD pathology [9–11]. Here, we also found that long-term metformin treatment causes learning impairment in AD mice.

We observed that metformin had different effects on cognition between young and old or AD conditions in mice. AMPK activation is generally considered beneficial for metabolism and cognition [50, 51]. However, some studies have suggested that overactivation of AMPK in aging or pathological conditions could be detrimental [52–54]. These may explain that metformin, an AMPK activator, exerted different outcomes according to the presence or absence of aging and AD pathology.

However, we did not see significant changes in the levels of p-AMPK/AMPK upon metformin treatment. This might result from homeostatic adaptation after chronic treatment as tight regulation of AMPK activity would be crucial in the brain owing to the potentially detrimental consequences of its overactivation [52, 54]. More likely, it might have been a matter of timing between the maximal drug action in vivo and brain tissue sampling. Even though we only examined the hippocampal tissues in AD mice, target engagement of metformin may vary across the brain regions and different pathologic conditions (young vs. old age or normal aging vs. AD). As we assessed multi-domain cognition, a future study should examine region-specific and disease-specific effects of metformin to elucidate the relationship between metformin-induced cognitive changes and functional changes in different brain regions.

Instead, we found that metformin led to an increase in AMPKα1-subunit expression. Interestingly, the increased expression of the AMPKα1-subunit has been reported in human and mouse AD brains while experimental suppression of AMPKα1 improved cognition in AD model mice [35]. Thus, elevated levels of AMPKα1 may account for metformin-induced learning impairment in our old and AD mice, although further research is needed for this topic.

The strength of this study is the use of touchscreen-based tasks for multi-domain cognitive assessment. This thorough behavioral approach can be considered to enhance the reliability and translational value of our research outcomes [55]. However, there are also limitations in this study. First, the confounded effects on cognition in NT mice could not be completely excluded, as we conducted the same behavioral assessment twice on the same cohort. However, as the behaviors of both groups were compared under the same re-test condition, we assume that our findings are reliable. Second, we used food-restricted male AD mice in behavioral experiments and the littermate female mice that were fed *ad libitum* for molecular studies. We did this to avoid the confounding effects of food restriction on AMPK activity while minimizing the number of animals needed to be bred. However, as sex difference is well known in AD [56, 57], sex-specific effects of metformin should be further explored. Indeed, a previous study showed that metformin causes cognitive impairment in male AD mice but enhances cognition in female mice [58]. Third, mechanistic studies are further required to elucidate molecular pathways through which metformin exerts beneficial or unfavorable effects on cognition and AD pathology including the association of AMPK activity and subunit expression. As we did not compare AMPK signaling between young and old NT mice, it remains to be clarified whether the behavioral outcome in old age was directly related to altered AMPK activity or mediated by other pathways involved in the chronic action of metformin. In addition, it remains unclear whether the chronic effects of metformin identified in our study are related to the age of the mice, the treatment period, or both. Lastly, we found that the survival rate was slightly, though not significantly, higher in metformin-treated versus vehicle-treated AD male mice (Fig. 3B). This finding may be in line with previous findings showing that AMPK activation increases life span in animals [59, 60]. The possibility of survival bias should be stressed as AD mice that lived longer due to metformin treatment might have performed worse in the PAL task. However, it is unlikely that the survival bias can solely account for the poor performance of metformin-treated AD mice as we also observed metformin-induced aggravation of AD pathologies in female AD mice, that showed no difference in life span (Fig. 4).

In conclusion, we found that long-term treatment of metformin led to cognitive impairment in mice at old age as well as in the AD model mice despite enhanced cognition observed in NT mice at young age. Chronic metformin treatment also exacerbated AD pathologies such as increased levels of Aβ and p-tau and increased expression of GSK3β and AMPKα1 in the hippocampus of AD mice. Therefore, we argue that drug repurposing of metformin should be carefully reconsidered, especially when it is intended for individuals with AD.

### Supplementary information

Supplementary information

## Data Availability

All materials, data, and associated protocols are available from the corresponding author upon reasonable request.
