# Supplementary material for: Reconsidering repurposing: long-term metformin treatment impairs cognition in Alzheimer’s model mice
Source: Transl Psychiatry. 2024 Jan 18;14:34. doi: 10.1038/s41398-024-02755-9 (PMC10796941; doi:10.1038/s41398-024-02755-9)
Supplement: Supplementary file 1 — Supplementary information [file 41398_2024_2755_MOESM1_ESM.docx]

**Reconsidering repurposing: Long-term metformin treatment impairs cognition in Alzheimer’s model mice**

So Yeon Cho, MS^1,2,3^, Eun Woo Kim, MS^4,5†^, Soo Jin Park, PhD^6,7†^, Benjamin U. Phillips, PhD^8^ , Jihyeon Jeong, PhD^1,2,3^, Hyunjeong Kim, PhD^2,3^, Christopher J. Heath, PhD^9^, Daehwan Kim, BS^6^, Yurim Jang, MS^10^, Laura López-Cruz, PhD^9^, Lisa M. Saksida, PhD^11,12^, Timothy J. Bussey, PhD^11,12^, Do Yup Lee, PhD^6,7,10^, Eosu Kim, MD, PhD^1,2,3,4*^

^1^Graduate School of Medical Science, Brain Korea 21 Project, Yonsei University College of Medicine, Seoul 03722, Republic of Korea

^2^Department of Psychiatry, Laboratory for Alzheimer’s Molecular Psychiatry, Institute of Behavioral Science in Medicine, Yonsei University College of Medicine, Seoul 03722, Republic of Korea

^3^Metabolism-Dementia Research Institute, Yonsei University College of Medicine, Seoul 03722, Republic of Korea

^4^Graduate School of Medicine, Yonsei University, Seoul 03722, Republic of Korea

^5^Department of Nursing, Seoyeong University, Gwangju 61268, Republic of Korea

^6^Department of Agricultural Biotechnology, Seoul National University, Seoul 08826, Republic of Korea

^7^Research Institute for Agricultural and Life Sciences, Seoul National University, Seoul 08826, Republic of Korea

^8^Department of Psychology, The University of Cambridge, Cambridge CB2 3EB, United Kingdom

^9^School of Life, Health and Chemical Sciences, The Open University, Milton Keynes MK7 6AA, United Kingdom

^10^Interdisciplinary Program in Agricultural Genomics, Center for Food and Bioconvergence, Seoul National University, Seoul 08826, Republic of Korea

^11^Robarts Research Institute, Schulich School of Medicine and Dentistry, Western University, London N6A 5K8, Canada

^12^Department of Physiology and Pharmacology, Schulich School of Medicine and Dentistry, Western University, London N6A 5C1, Canada

^†^E.W.K. and S.J.P. contributed equally to this work.

*Corresponding to Eosu Kim, MD, PhD

Department of Psychiatry,

Yonsei University College of Medicine,

Seoul 03722, Republic of Korea

Phone: +82-2-2228-1629

Email: eosu.kim@yonsei.ac.kr

**Supplementary Materials and Methods**

**Cell culture and transfection**

Neuro2a (mouse neuroblastoma cell line) was purchased from the American Type Culture Collection (ATCC; Manassas, VA, USA). Neuro2a-APP_695_ stable cell line was generated from our lab. Neuro2a transfected with pcDNA 3.1-APP_695_ vector was selected by incubating with geneticin (200 μg/ml, Gibco, Grand Island, NY, USA) for 48 h. Selected cells were sub-cultured in DMEM-OptiMEM (Gibco) supplemented with 10% fetal bovine serum (FBS; Hyclone Laboratories, Inc., Logan, UT, USA), 1% penicillin/streptomycin (PS; Gibco) and 200 μg/ml geneticin and stored at -80 ℃ until further experiment. Neuro2a and Neuro2a-APP_695_ were maintained with high-glucose Dulbecco’s modified Eagle medium (Hyclone) supplemented with 10% FBS and 1% PS in a humidified incubator at 37 ℃ with 5% CO2.

**Mouse primary neuron culture**

Pregnant female C57BL/6 mice were purchased from Koatech (Pyeongtaek, Korea). After deeply anesthetized mice, E17 embryos were harvested from the uterus and transferred to an ice-cold dissection medium containing HBSS without calcium and magnesium (Gibco), 10 mM HEPES, 100 U/ml PS. In a fresh ice-cold dissection medium, the cortices and hippocampi were dissected. Chopped tissues were dissociated by chopping solution (140 U/ml DNase, 0.05% Trypsin-EDTA in dissection medium) at 37°C for 15 min and transferred to dissection medium. After centrifuging at 1,000 rpm and RT for 5 min, cells in culture medium containing neurobasal medium (Gibco), B-27 supplement (1:50; Gibco), 2 mM Glutamax (Gibco), 100 U/ml PS plated onto 12-well plate pre-coated with poly-D-lysine (Sigma). The whole medium was changed after 2 h incubation. The half of medium was replaced with fresh culture medium without PS every 3-4 days.

**Supplementary animals**

Male db/db mice were purchased from Center Lab. Animal Inc. (Seoul, Korea) and were treated with 2 mg/ml metformin for 7 days after the random group distribution which kept blinded (n = 3 per group). All mice were housed in a specific pathogen-free room with a 12–h light/dark cycle (lights on from 8:00 a.m. to 8:00 p.m.) and humidity- and temperature-controlled environment. All procedures were approved by Yonsei University Health System Institutional Animal Care and Use Committee (IACUC) and performed in accordance with National Institute of Health guidelines for the Care and Use of Laboratory Animals. The sample size had been estimated to ensure adequate power to detect a pre-specified effect size.

**Experimental cohort**

C57BL/6 and 3xTg-AD mice were divided into 3 different cohorts with the random group distribution which kept blinded: male C57BL/6 mice with food restriction (cohort 1: vehicle group, n = 16; metformin group, n = 16), male 3xTg-AD mice with food restriction (cohort 2: vehicle group, n = 14; metformin group, n = 12), and female 3xTg-AD mice without food restriction (cohort 3; vehicle group, n = 6; metformin group, n = 5). For cohorts 1 and 2, water was provided *ad libitum* throughout the experiment, while food and water were provided *ad libitum* to cohort 3. The mice of cohorts 1 and 2 were used for the behavioral experiments. Their weight was maintained at 85–90% of their average free-feeding weight. All behavioral experiments were conducted once a day for 5–7 days a week. Cohort 3 was used for the molecular experiments. For each behavioral task, the mice that did not achieve the performance criteria were excluded. Due to the hippocampal shrinkage, one mouse in the vehicle group was excluded from western blot analysis, and one mouse in the metformin group was excluded from immunohistochemistry analysis. The sample size had been estimated to ensure adequate power to detect a pre-specified effect size.

**Apparatus**

All behavioral training and test sessions were conducted in standard Bussey–Saksida mouse touchscreen chambers (Campden Instruments Ltd., Loughborough, UK) for 5–7 days a week described elsewhere [1]. The apparatus consisted of four standard modular testing chambers housed within a sound- and light-attenuating box (Campden Instruments Ltd.). A 3-W house light, tone generator, ventilating fan, and an IR camera are present in the box. The trapezoidal-shaped testing chamber consisted of a metal frame, Perspex sidewalls, and a stainless-steel grid floor (20 × 18 × 24 cm, Campden Instruments Ltd.). A touch-sensitive screen (12.1-inch; resolution, 800 × 600) and a reward receptacle (magazine) with a 3-W light bulb for illumination and an infrared beam for head entry detection were fitted at the front and rear sides of the chamber, respectively. Infrared beams were assembled around the testing chamber to detect the subjects’ movements. The front infrared beam was 6 cm away from the screen, and the rear beam was 3 cm away from the magazine. The magazine was attached to a reward dispenser filled with the strawberry milkshake (Seoul Strawberry Milk®; SeoulMilk Dairy Cooperative, Seoul, Republic of Korea) and provided 20 µL of reward per trial. In front of the touchscreen, a black Perspex mask was placed. This study used three different masks owing to the task specificity. For the FR, PR, and 5-CSRT tasks, a 5-window mask was used. For the PAL task, a 3-window mask was used. For the VD and reversal tasks, a 2-window mask was used. ABET II Touch software and Whisker Vehicle software (Campden Instruments Ltd.) were used to control the system and collect data.

**Shaping**

Before conducting the behavioral assessment, the mice were acclimated to the facility and the researcher for 1 week. Pretraining started after food restriction and was conducted as previously described [1-3]. The mice were habituated to the testing chamber environment for 20 min for at least 2 days. Before the onset of each session, 200 μL of reward was manually placed in the magazine aperture. When all mice consumed the reward for 2 consecutive days, they were subjected to the pretraining sessions. The mice were then trained for the initial touch training (ITT) to associate the stimuli-off on the screen with the reward. The white square stimulus was presented in the middle of the 5-window mask. After 30 s, the stimulus disappeared with the tone, and the 20-μL reward was delivered with the magazine illumination. When the mice collected the reward by putting their head into the magazine, the subsequent trial commenced after a 5-s inter-trial interval (ITI). If the mice emitted a response to the stimulus prior to offset, a triple amount, 60 μL, of reward was delivered. After the mice completed 30 trials within 60 min for 1 day, they were subjected to must-touch training (MTT) to associate the response to the stimulus with the delivery of the reward. The mice had to touch the stimulus to earn 20 μL of the reward. In this training, a 5-s ITI was provided between trials. When the mice completed 30 trials within 60 min for 1 day, they were subjected to the FR and PR tasks. After the FR and PR tasks, the mice were trained for must-initiate training (MIT) to initiate trials by nose-poking the magazine to break the infrared beam. The stimulus randomly appeared on one window of the 5-window mask. After touching the stimulus and reward collection, the magazine was illuminated after 5 s of ITI, which indicated the onset of the new trial. The mice had to complete 30 trials within 60 min for 2 consecutive days.

**Fixed ratio (FR) and progressive ratio (PR) schedule**

The FR and PR task procedures were adapted from the general procedure described elsewhere [4-6]. After the mice completed the series of pretraining sessions, they performed the FR task. The mice had to perform the FR task within 60 min. The stimulus was presented in the middle of the 5-window mask until the mice responded (Fig. 1C). Between the trials, 4.5 s of ITI was provided. At first, the mice performed FR1 sessions for 30 trials, requiring one screen touch to get a single reward with the tone and magazine illumination. After completing FR1 sessions for 1 day, the mice were subjected to FR2 (15 trials, two screen touches for a single reward), FR3 (10 trials, three screen touches for a single reward), and FR5 (30 trials, five screen touches for a single reward) sessions in the given order. To prevent overtraining and match the acquisition levels of all mice, they received a reminder session once a week when one mouse accomplished the criterion faster than other mice. When all mice completed the FR5 for 2 consecutive days, the baseline was set with FR5 sessions for 2 consecutive days. After setting the baseline, the mice were subjected to FR5-uncapped (FR5-UC) sessions. These sessions lasted for 60 min without the limit of trial numbers. FR5-UC sessions were conducted on two separate days by matching the weight of the mice between sessions. The mice performed the PR 4 sessions for 3 consecutive days after the FR task. The number of screen touches to get a single reward was gradually increased by 4 (e.g., 1, 5, 9, 13, …) in the PR4 sessions. Each session of the PR task was terminated either within 60 min or 5 min without any movement.

**5-Choice serial reaction time (5-CSRT) task**

The 5-CSRTT task procedure was adapted from the general procedure described elsewhere [2, 7]. After completing the MIT, the mice performed the 5-CSRT task for 60 trials within 60 min per day. The stimulus was presented in one window of the 5-window mask in a pseudo-random manner (Fig. 1F). Each trial began with the illumination of the magazine. The mice nose-poked the magazine for initiating the trial. After 10 s of delay, the mice had to respond to the stimulus during stimulus duration (SD) and limited hold (LH). When the mice touched the stimulus or blank window, it was recorded as an accurate response (correct for the stimulus and incorrect for the blank window). When they did not touch the screen, it was recorded as an omission. When the mice touched the screen during the delay period, it was recorded as a premature response. When the mice touched the screen after touching the stimulus or blank window, it was recorded as a perseverative response. After responding to the stimulus, the reward was delivered with tone and magazine illumination. However, after touching the blank window or omitting the response, the 10-s time-out period with house light extinguishment was commenced as punishment. There was a 20-s ITI between each trial. The SD was initially set to 32 s, and the following LH was set to 7 s. When the mice accomplished the criterion of the 5-CSRT task (completion of 60 trials within 60 min; for 6-month-old C57BL/6 and 8-month-old 3xTg-AD mice: accuracy ≥80% and omission ≤20% for 2 consecutive days; for 22-month-old C57BL/6 mice: accuracy ≥75% and omission ≤25% for 2 consecutive days) on each SD, they were subjected to sessions with gradually reducing SDs (16 s, 8 s, 4 s, and 2 s). To prevent overtraining and match the acquisition levels of all mice, they received a reminder session once a week when one mouse accomplished the criterion faster than other mice. When all mice completed the criterion in 2 s SD, the baseline was set at 2 s SD for 2 consecutive days. After setting the baseline, the mice performed a within-session probe test for 4 consecutive days, at 60 trials within 60 min per day. Four different SDs (2.0 s, 1.5 s, 1.0 s, and 0.5 s) were presented in a pseudo-random manner to increase attentional demands.

**Paired associates learning (PAL) task**

The PAL task procedure was adapted from the general procedure described elsewhere [8]. The dPAL task was initially set to 24 trials, and the number of trials was gradually increased to 72 trials (24, 36, 48, 60, and 72) when all mice completed the designated trials for 2 consecutive days. The mice performed the task with the designated trials for 60 min per day. In the dPAL task, one of six trial types with two different line-shaped stimuli was pseudo-randomly presented on the screen (Fig. 1L). On the 3-window mask, one location showed the correct stimuli (S+) that matched the location, another showed incorrect stimuli (S-), and the other remained blank. Each trial started with the illumination of the magazine. The mice nose-poked the magazine for initiating the trial. The stimuli were presented without a delay period. When the mice responded to the correct stimulus, the reward was delivered with the tone and the magazine illumination. The next trial commenced after a 15-s ITI. However, when the mice responded to the incorrect stimulus, a 5-s time-out period with house light extinguishment and a 5-s ITI were given, and correction trials (revision-like trials) commenced with the same stimuli combination that the mice were incorrect until the mice responded to the correct stimulus. When the group’s average accuracy was higher than 80% (for 12-month-old C57BL/6 and 11-month-old 3xTg-AD mice) or 75% (for 22-month-old C57BL/6 mice), they were moved to the sPAL task. In the sPAL task, the same stimulus was presented on two spatially different windows (Fig. 1O). One location was correct (S+), while the other was incorrect (S+). After performing the dPAL task (60 trials) without the correction trials for 2 consecutive days within 60 min, the mice performed the sPAL task (60 trials) without the correction trials for 3 consecutive days; each trial had to be completed within 60 min. The purpose of the sPAL task is to elucidate whether the mice performed the dPAL task with object-location associative memory or conditional rule such as “if AC, select the left; if BC, select the right.” sPAL retention sessions were conducted once a week for 3 weeks after the sPAL task.

**Visual discrimination (VD) and reversal task**

The VD and reversal task procedures were adapted from the general procedure described elsewhere [9-11]. The mice performed 30 trials within 30 min per day. On the 2-window mask, two different stimuli were presented pseudo-randomly (Fig. 2L). Regardless of the location, one stimulus was correct (S+), and the other was incorrect (S-). When the mice touched the correct stimulus, the reward was delivered with tone and magazine illumination. However, when they touched the incorrect stimulus, a 5-s time-out period and house light extinguishment were given, and correction trials were commenced until the mice responded to the correct stimulus. There was a 20-s ITI between each trial. When the mice achieved the criterion of the VD task (accuracy ≥80% for two consecutive days), the baseline was set for 2 consecutive days. To prevent overtraining and match the acquisition levels of all mice, when one mouse accomplished the criterion faster than others, they received a reminder session once a week. After the baseline was set with the VD task, the mice performed the reversal task. In this task, the correct (S+) stimulus of the VD task was reversed to incorrect (S-), and the incorrect (S-) stimulus of the VD task was reversed to correct (S+) (Fig. 2O). Except for the stimuli shift, the process of the reversal task was identical to the VD task. When a mouse achieved the criterion of reversal task (accuracy ≥80% for 2 consecutive days), it was subjected to the reversal retention session. Each mouse performed one retention session 10 days after the last reversal session.

**Western blot sampling**

For mouse hippocampal tissue sampling, dissected hippocampi were homogenized with lysis buffer containing 20 mM Hepes (pH 7.0), 1 mM EDTA, 1 mM EGTA, 10 mM KCl, 1.5 mM MgCl2, 250 mM sucrose, phosphatase inhibitors (1mM NaF, 1mM Na3VO4, 1.15mM Na2MoO4, 2mM C3H2N4, 4mM C4H4Na2O6∙2H2O), and protease inhibitors (Complete, EDTA-Free Protease Inhibitor Cocktail; Roche, Indianapolis, IN, USA) at a concentration of 10 µL per 1 mg wet weight of hippocampal tissue. The homogenates were incubated for 15 min on ice with vortexing every 2–3 min and centrifuged at 8,000 ×g and 4°C for 30 min. After collecting the supernatants from the homogenates, the concentrations of proteins were quantified using DC assay (Bio-Rad Laboratories, Hercules, CA, USA) to make loading samples with 20 µg protein each. For cell sampling, cells were lysed in ice-cold RIPA buffer with phosphatase inhibitor and protease inhibitor (Roche). Total lysates were centrifuged at 12,000 rpm for 20 minutes at 4℃. After collecting the supernatants, the protein concentration of the cell lysates was determined by the BCA Protein assay kit (Thermo Fisher Scientific, MA, Waltham, USA) to make loading samples with 20 µg protein each. For cultured mouse primary neuron sampling, cells were harvested with 120 µL Laemmli buffer per well on days in vitro (DIV) 18. All loading samples were boiled at 95°C for 5 min.

**Western blot analysis**

Samples were separated on electrophoresis using 8% or 10% SDS-polyacrylamide gel at 70 V for 3.5 h and transferred onto polyvinylidene difluoride (PVDF) membranes (Merck Millipore, Burlington, MA, USA) at 30 V for 16 h. The membranes were washed with tris-buffered saline (TBS) containing 0.1% Tween-20 (TBST), and nonspecific binding was blocked with 5% skim milk in TBST for 60 min at room temperature (RT). After blocking, the membranes were incubated with primary antibodies (1:1,000–1:5,000) overnight at 4°C. After washing with TBST, the membranes were incubated with secondary antibodies and conjugated with horseradish peroxidase (1:5,000–1:10,000) for 60 min at RT. After washing with TBST, enhanced chemiluminescence solution (Amersham™ ECL™ western blotting detection reagent; GE Healthcare, Piscataway, NJ, USA) was applied to the membranes to detect target protein bands using a luminescent image analyzer (ImageQuant LAS 4000 mini; GE Healthcare). The intensities of detected target protein bands were normalized to that of β-actin or GAPDH. The antibodies used are listed in Supplementary Table 2.

**Immunohistochemistry**

The brain hemispheres were freshly embedded with an optimal cutting temperature (OCT) compound in a cryomold (Sakura Finetek, Torrance, CA, USA) and frozen. Fresh frozen brains were cut into 15-µm coronal sections on a cryostat (Leica biosystems Buffalo Grove, IL, USA). The sections containing the cortex and hippocampus were immediately placed on gelatin-subbed slides and stored at -80°C until further analysis. The sections were airdried overnight and fixed with phosphate-buffered saline (PBS) containing 4% paraformaldehyde (PFA) for 10 min at RT. The sections were permeabilized with PBS containing 0.3% Triton X-100 for 60 min and blocked with PBS containing 5% bovine serum albumin (BSA) and 0.3% Triton X-100 for 1 h. The sections were incubated with an antibody against D56D2 (1:100; Cell Signaling Technology, Danvers, MA, USA) diluted in PBS containing 2% BSA and 0.3% Triton X-100 overnight at 4°C. After washing with PBS, the sections were incubated with a secondary antibody (1:100; Goat-anti-rabbit; Jackson Immunoresearch Lab, Inc., West Grove, PA, USA) diluted in PBS containing 2% BSA for 60 min at RT. All sections were washed with PBS and counterstained with 6-diamidino-2-phenylindole (DAPI; 1:2,000; Sigma-Aldrich) in PBS. The slides were mounted with Vectashield mounting medium (Vector Lab, Inc., Burlingame, CA, USA) and covered by a coverslip. The immunoreactivity was observed under a fluorescence microscope (Olympus, Tokyo, Japan). The antibodies used are listed in Supplementary Table 2.

**Immunocytochemistry**

On DIV 18, mouse primary neurons grown in the coverslips were washed with ice-cold PBS twice and fixed with 4% PFA for 20 min. The cells were permeabilized with PBS containing 0.15% Triton X-100 for 30 min and blocked with PBS containing 5% BSA for 1 h. The cells were incubated with an antibody against synaptophysin, PSD-95, NeuN, and MAP2 (1:500) diluted in PBS containing 2% BSA overnight at 4°C. After washing with PBS, the cells were incubated with secondary antibodies (1:500; Donkey-anti-rabbit, Donkey-anti-mouse, Donkey-anti-guinea pig; Jackson Immunoresearch Lab, Inc.) diluted in PBS containing 2% BSA for 60 min at RT. All cells were washed with PBS, counterstained with DAPI in PBS, and mounted with Vectashield mounting medium. The immunoreactivity was observed under a fluorescence microscope. The antibodies used are listed in Supplementary Table 2.

**Aβ ELISA**

The quantitative assessment of Aβ1-42 of secretion was measured using the High sensitivity Human Amyloid β42 ELISA Kit (EMD Millipore Corp., Burlington, MA, USA) according to the manufacturer`s instructions. After treating Neuro2a-APP_695_ with metformin for 24 h, 100 μl of culture medium was used. All results are normalized with the protein concentration of the cell extracts determined with the BCA protein Assay Kit.

**Blood glucose concentration measurement**

Blood samples were collected by cutting ~1 mm off the tip of the mouse tail. Blood glucose concentration was twice measured using the glucometer system Accu-Chek Performa kit (Roche Diagnostics GmbH, Mannheim, Germany) before the onset and after the end of the behavioral experiment.

**Serum extraction**

Whole blood was collected from the anesthetized mice by heart puncture. Blood samples were transferred to BD Microtainer tubes (Becton, Dickinson and Company, NJ, USA) and left in RT for 30 min to clot. Then, the samples were centrifuged at 1300 ×g for 30 min. The serum left above the polymer barrier was harvested into a new tube. The serum samples were stored at -80℃ until further analysis.

**Metabolomic analysis**

The concentration of metformin in mouse serum was measured via Liquid chromatography–mass spectrometry (LC-MS). To detect metformin in the serum, 1400 µL of extraction solvent (methanol:isopropanol:distilled water=3:3:2, v/v/v) was added to 50 µL of the serum sample. The solutions were kept on ice and sonicated for 15 min. After centrifugation at 13,200 rpm and 4℃ for 15 min, the supernatants of samples were collected into new tubes. The samples were dehydrated and concentrated using a ScanVac speed vacuum concentrator (Labogene, Seoul, Republic of Korea). The extracts were stored at -80℃ until further analysis. The dried samples were reconstituted with 250 µL of distilled water containing 0.1% formic acid for LC-Orbitrap MS analysis. Chromatographic separation was performed using an Ultimate-3000 UPLC system (Thermo Fisher Scientific, MA, USA) and an Acquity UPLC BEH C18 Column (1.7 µm; 100 mm × 2.1 mm; Waters, MA, USA) equipped with a UPLC BEH HILIC VanGuard pre-column (1.7 μm; 5.0 mm × 2.1 mm; Waters). The mobile phase consisted of two different buffers: buffer A (0.1% formic acid in distilled water) and buffer B (0.1% formic acid in acetonitrile). The flow rate was set to 0.3 mL/min, and the gradient of buffer B phase was programmed as follows: 0–0.1 min, 0.5% B; 10 min, 80% B; 10.1–12 min, 99.5% B; 12.1–15 min, 0.5% B. Mass-spectrometric analysis was performed using a Q-Exactive plus instrument (Thermo Fisher Scientific) with positive ionization mode for detection. The acquisition method was conducted using a Full MS scan ranging from 120 to 1800 m/z. Data acquisition and preprocessing were performed using Xcalibur software (Thermo Fisher Scientific). For peak alignment and annotation, raw data files were processed using MS-DIAL version 4.90 [12]. PCA was performed using the SIMCA 17 software (Umetrics AB, Umea, Sweden). HCA and metabolite set enrichment analysis (MSEA) were conducted using MetaboAnalyst 5.0 (http://metaboanalyst.ca) [13]. HCA was based on the Ward clustering algorithm with Euclidean distance measure. MSEA used overrepresentation analysis with Small Molecular Pathway Database (SMPDB). PERMANOVA and multiple linear regression were performed in R version 4.2.1 and RStudio version 2022.02.3. The following R packages were used: Vegan package for PERMANOVA and Maaslin2 package for multiple linear regression. The full names of analyzed metabolites are shown in Supplementary Table 3.

**Supplementary data statistical analysis**

Statistical analyses of behavioral and molecular data, except metabolomics data, were conducted using R version 3.6.3 and GraphPad Prism version 9 (Graphpad Software Inc., La Jolla, CA, USA). Comparison between the two or more than three groups' differences was analyzed by t-test and Dunnett’s test, respectively. Repeated measured data were evaluated by repeated-measures (RM) analysis of variance analysis (ANOVA; when the number of groups was the same) or mixed effects model (when the number of groups was different). All data were expressed as the mean ± SEM. Significance was set at α < 0.05.

**Supplementary References**

1. Horner AE, Heath CJ, Hvoslef-Eide M, Kent BA, Kim CH, Nilsson SR, et al. The touchscreen operant platform for testing learning and memory in rats and mice. Nat Protoc 2013;8(10):1961-84.

2. Mar AC, Horner AE, Nilsson SR, Alsiö J, Kent BA, Kim CH, et al. The touchscreen operant platform for assessing executive function in rats and mice. Nature protocols 2013;8(10):1985-2005.

3. Oomen CA, Hvoslef-Eide M, Heath CJ, Mar AC, Horner AE, Bussey TJ, et al. The touchscreen operant platform for testing working memory and pattern separation in rats and mice. Nat Protoc 2013;8(10):2006-21.

4. Heath CJ, Phillips BU, Bussey TJ, Saksida LM. Measuring Motivation and Reward-Related Decision Making in the Rodent Operant Touchscreen System. Curr Protoc Neurosci 2016;74:8 34 1-8 20.

5. Kim EW, Phillips BU, Heath CJ, Cho SY, Kim H, Sreedharan J, et al. Optimizing reproducibility of operant testing through reinforcer standardization: identification of key nutritional constituents determining reward strength in touchscreens. Mol Brain 2017;10(1):31.

6. White MA, Kim E, Duffy A, Adalbert R, Phillips BU, Peters OM, et al. TDP-43 gains function due to perturbed autoregulation in a Tardbp knock-in mouse model of ALS-FTD. Nat Neurosci 2018;21(4):552-63.

7. Romberg C, Mattson MP, Mughal MR, Bussey TJ, Saksida LM. Impaired attention in the 3xTgAD mouse model of Alzheimer's disease: rescue by donepezil (Aricept). J Neurosci 2011;31(9):3500-7.

8. Kim CH, Heath CJ, Kent BA, Bussey TJ, Saksida LM. The role of the dorsal hippocampus in two versions of the touchscreen automated paired associates learning (PAL) task for mice. Psychopharmacology (Berl) 2015;232(21-22):3899-910.

9. Brigman JL, Feyder M, Saksida LM, Bussey TJ, Mishina M, Holmes A. Impaired discrimination learning in mice lacking the NMDA receptor NR2A subunit. Learn Mem 2008;15(2):50-4.

10. Graybeal C, Feyder M, Schulman E, Saksida LM, Bussey TJ, Brigman JL, et al. Paradoxical reversal learning enhancement by stress or prefrontal cortical damage: rescue with BDNF. Nat Neurosci 2011;14(12):1507-9.

11. Romberg C, Horner AE, Bussey TJ, Saksida LM. A touch screen-automated cognitive test battery reveals impaired attention, memory abnormalities, and increased response inhibition in the TgCRND8 mouse model of Alzheimer's disease. Neurobiology of aging 2013;34(3):731-44.

12. Tsugawa H, Cajka T, Kind T, Ma Y, Higgins B, Ikeda K, et al. MS-DIAL: data-independent MS/MS deconvolution for comprehensive metabolome analysis. Nature methods 2015;12(6):523-6.

13. Xia J, Wishart DS. MSEA: a web-based tool to identify biologically meaningful patterns in quantitative metabolomic data. Nucleic acids research 2010;38(suppl_2):W71-W7.

**Supplementary figure and legends**

**
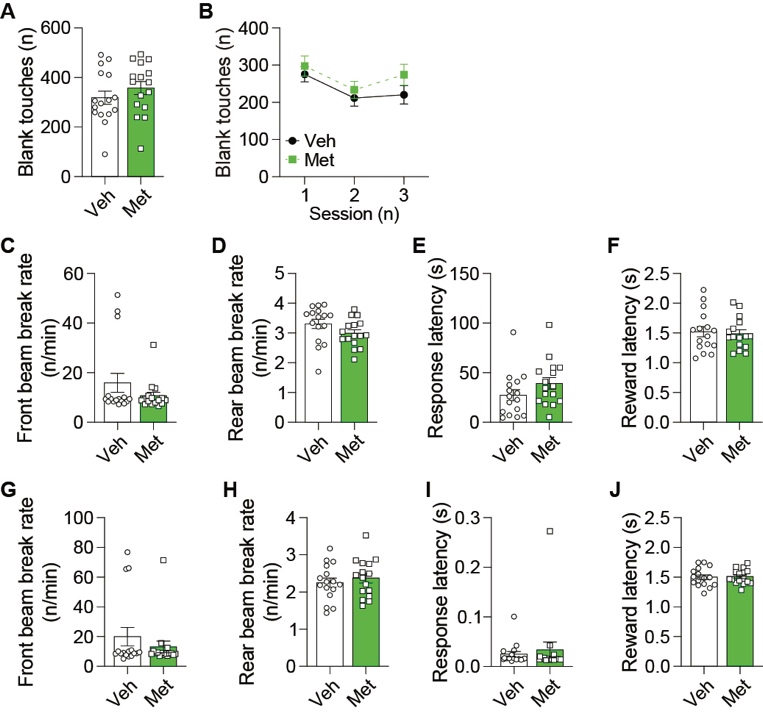
**

**Supplementary Figure 1.** Task performance and motor function of non-transgenic (NT) mice during the first FR and PR schedules. The number of blank touches of 4-month-old NT mice (Veh, n = 16; Met, n = 16) (A) in the FR schedule (*p* = 0.207; t-test) and (B) in the PR schedule (*p* = 0.240; RM-ANOVA). (C) Front beam break rate (*p* = 0.207), (D) rear beam break rate (*p* = 0.120), (E) response latency (*p* = 0.148), and (F) reward collection latency (*p* = 0.758; t-test) of 4-month-old NT mice in the FR schedule. (G) Front beam break rate (*p* = 0.357), (H) rear beam break rate (*p* = 0.495), (I) response latency (*p* = 0.614), and (J) reward collection latency (*p* = 0.870) of 4-month-old NT mice in the PR schedule. Data are presented as mean ± SEM. NT, non-transgenic C57BL/6 mice; Veh, Vehicle; Met, Metformin.

**
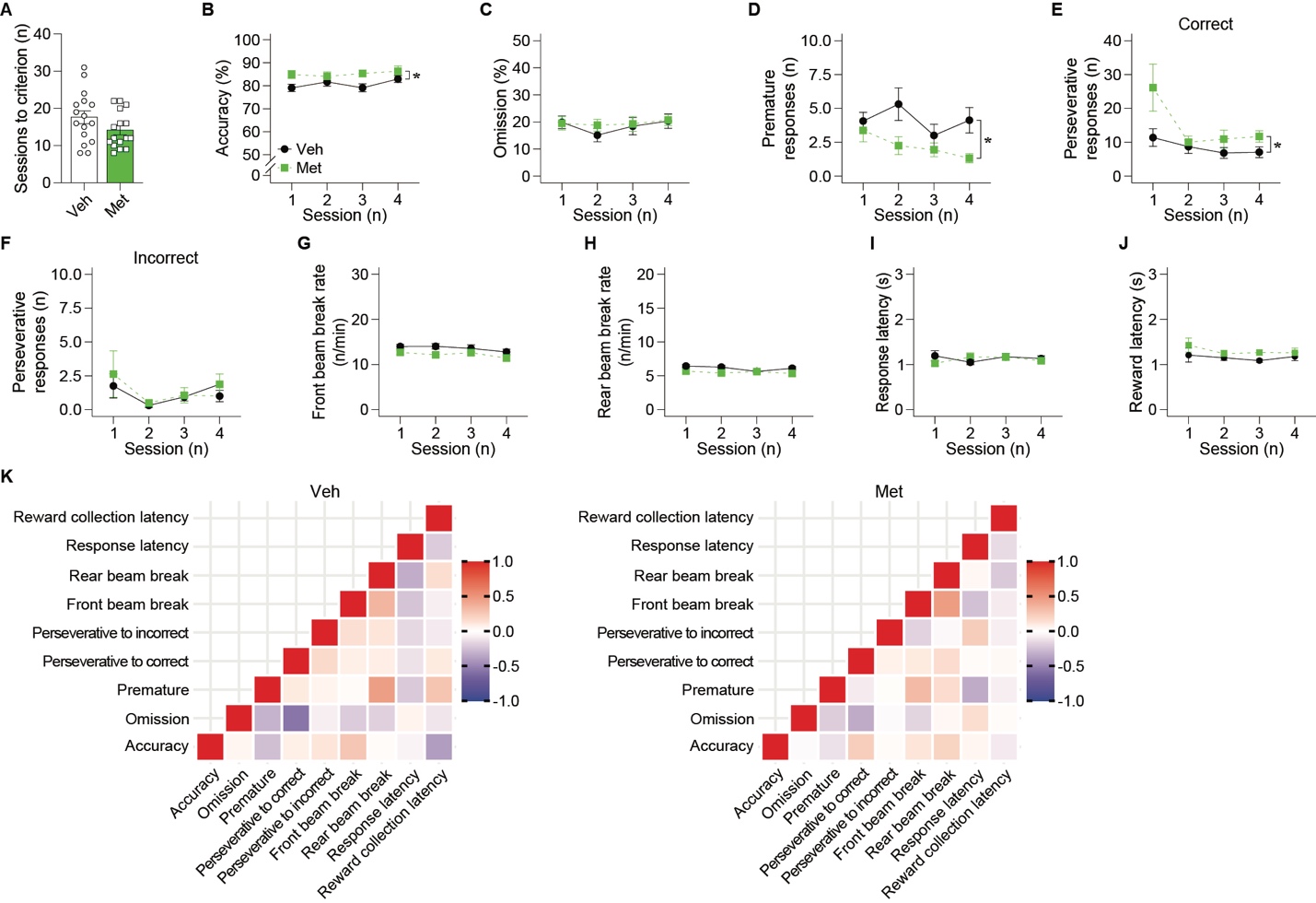
**

**Supplementary Figure 2.** Task performance and motor function of NT mice during the first 5-CSRT task. (A) The number of sessions required to accomplish the criterion of the 5-CSRT task during the acquisition phase of 5-month-old NT mice (Veh, n = 16; Met, n = 16; *p* = 0.107; t-test). (B) Accuracy (*p* = 0.017), (C) omission (*p* = 0.675), and the number of (D) premature responses (*p* = 0.026) and perseverative responses to (E) correct stimuli (*p* = 0.017) and (F) incorrect stimuli (*p* = 0.896; mixed effects model) of 6-month-old NT mice (Veh, n = 16; Met, n = 16) in each session of 5-CSRT probe test. (G) Front beam break rate (*p* = 0.087), (H) rear beam break rate (*p* = 0.081), (I) response latency (*p* = 0.694), and (J) reward collection latency (*p* = 0.129; mixed effects model) of 6-month-old NT mice in the 5-CSRT probe test. (K) Heatmap representing the relationship between the variables of the 5-CSRT task in Veh (left) and Met (right) groups of 6-month-old NT mice. Data are presented as mean ± SEM. **p* < 0.05 versus Veh. NT, non-transgenic C57BL/6 mice; Veh, Vehicle; Met, Metformin.

**
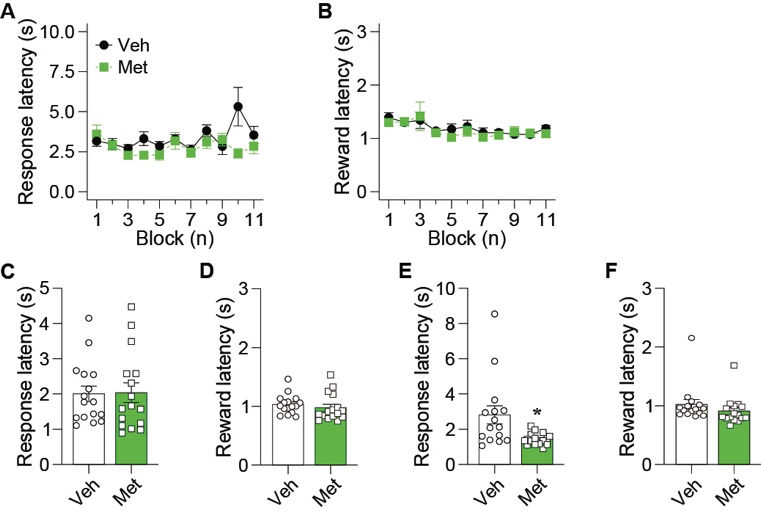
**

**Supplementary Figure 3.** Motor function of NT mice during the first PAL task. (A) Response latency (*p* = 0.177) and (B) reward collection latency (*p* = 0.553; mixed effects model) of 12-month-old NT mice (Veh, n = 16; Met, n = 13) in the dPAL task. The block consisted of approximately 300 trials (288–324) as the number of trials per session gradually increased. (C) Response latency (*p* = 0.923) and (D) reward collection latency (*p* = 0.434; t-test) of 16-month-old NT mice (Veh, n = 16; Met, n = 13) in the sPAL task. (E) Response latency (*p* = 0.012) and (F) reward collection latency (*p* = 0.275; t-test) of 16-month-old NT mice (Veh, n = 15; Met, n = 13) in the sPAL retention sessions. Data are presented as mean ± SEM. **p* < 0.05 versus Veh. NT, non-transgenic C57BL/6 mice; Veh, Vehicle; Met, Metformin.


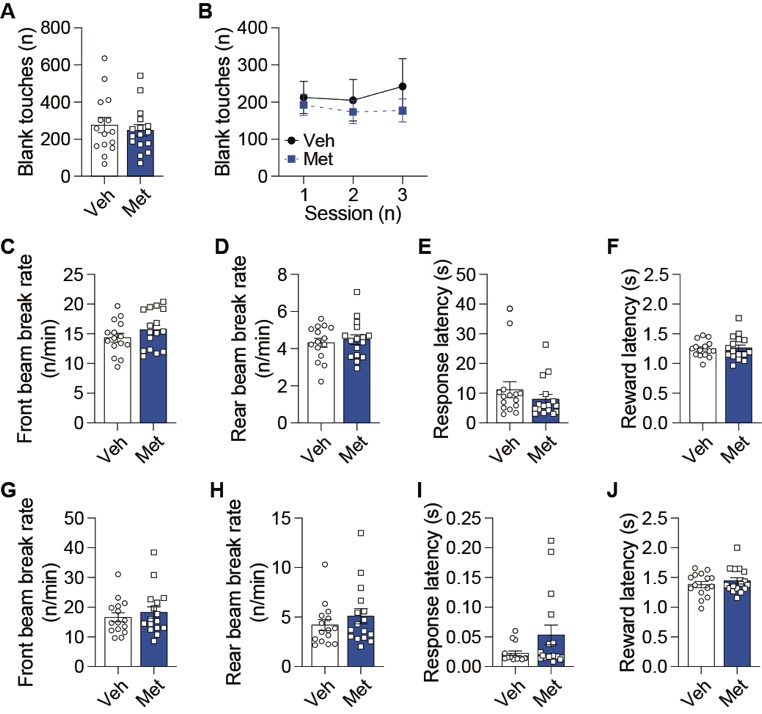


**Supplementary Figure 4.** Task performance and motor function of NT mice during the second FR and PR schedules. The number of blank touches of 20-month-old NT mice (Veh, n = 15; Met, n = 16) (A) in the FR schedule (*p* = 0.579; t-test) and (B) in the PR schedule (*p* = 0.534; RM-ANOVA). (C) Front beam break rate (*p* = 0.239), (D) rear beam break rate (*p* = 0.640), (E) response latency (*p* = 0.307), and (F) reward collection latency (*p* = 0.804; t-test) of 20-month-old NT mice in the FR schedule. (G) Front beam break rate (*p* = 0.505), (H) rear beam break rate (*p* = 0.351), (I) response latency (*p* = 0.087), and (J) reward collection latency (*p* = 0.414; t-test) of 20-month-old NT mice in the PR schedule. Data are presented as mean ± SEM. NT, non-transgenic C57BL/6 mice; Veh, Vehicle; Met, Metformin.

**
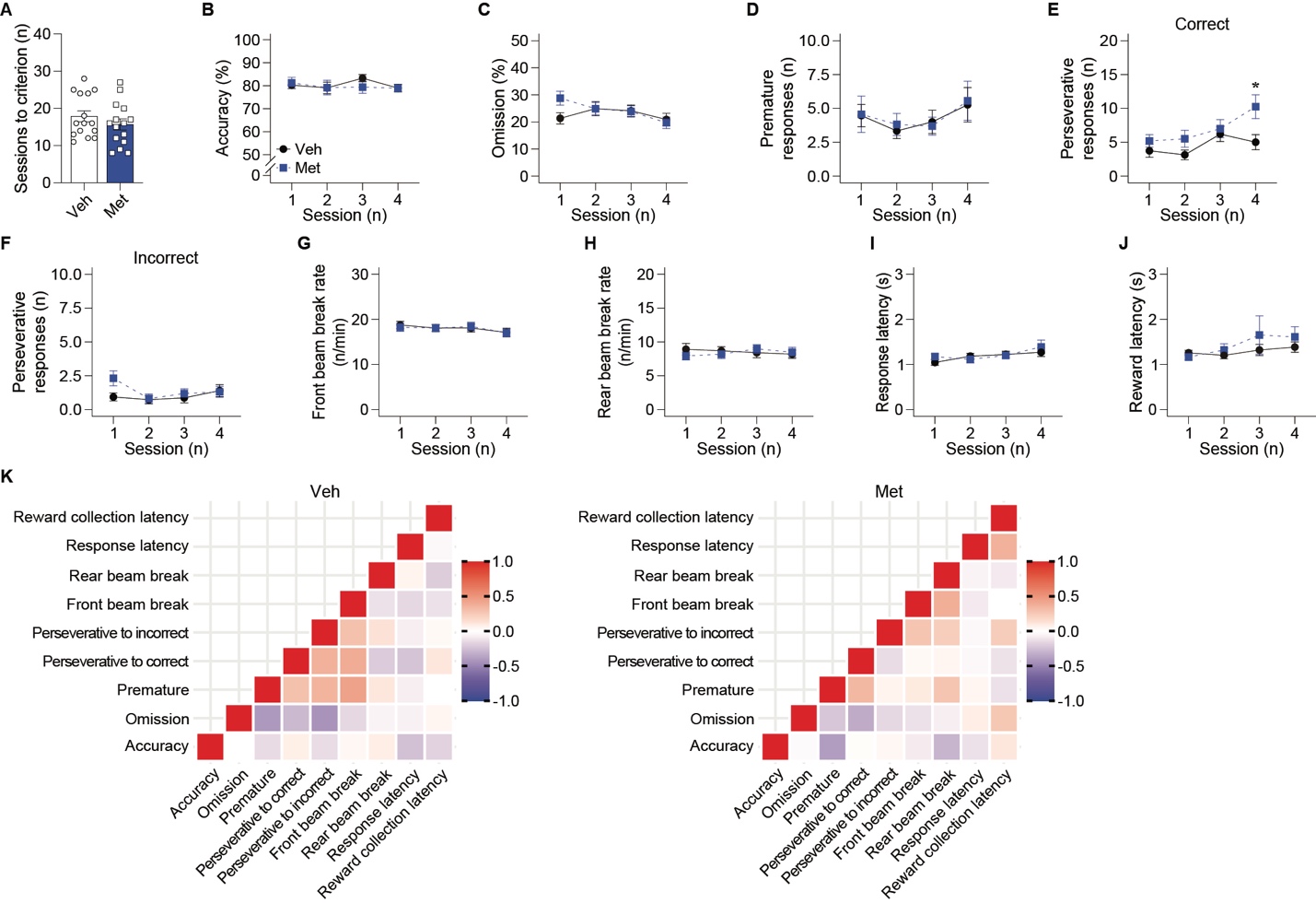
**

**Supplementary Figure 5.** Task performance and motor function of NT mice during the second 5-CSRT task. (A) The number of sessions required to accomplish the criterion of the 5-CSRT task during the acquisition phase of 20-month-old NT mice (Veh, n = 15; Met, n = 16; *p* = 0.350; t-test). (B) Accuracy (*p* = 0.771), (C) omission (*p* = 0.523), and the number of (D) premature responses (*p* = 0.889) and perseverative responses to (E) correct stimuli (Main effect of group, *p* = 0.076; group by session interaction, *p* = 0.047; simple effect of group in session 4, p = 0.019) and (F) incorrect stimuli (*p* = 0.286; mixed effects model) of 22-month-old NT mice (Veh, n = 15; MET, n = 16) in each session of 5-CSRT probe test. (G) Front beam break rate (*p* = 0.889), (H) rear beam break rate (*p* = 0.863), (I) response latency (*p* = 0.576), and (J) reward collection latency (*p* = 0.317; mixed effects model) of 22-month-old NT mice in the 5-CSRT probe test. (K) Heatmap representing the relationship between the variables of the 5-CSRT task in Veh (left) and Met (right) group of 22-month-old NT mice. Data are presented as mean ± SEM. **p* < 0.05 versus Veh. NT, non-transgenic C57BL/6 mice; Veh, Vehicle; Met, Metformin.

**
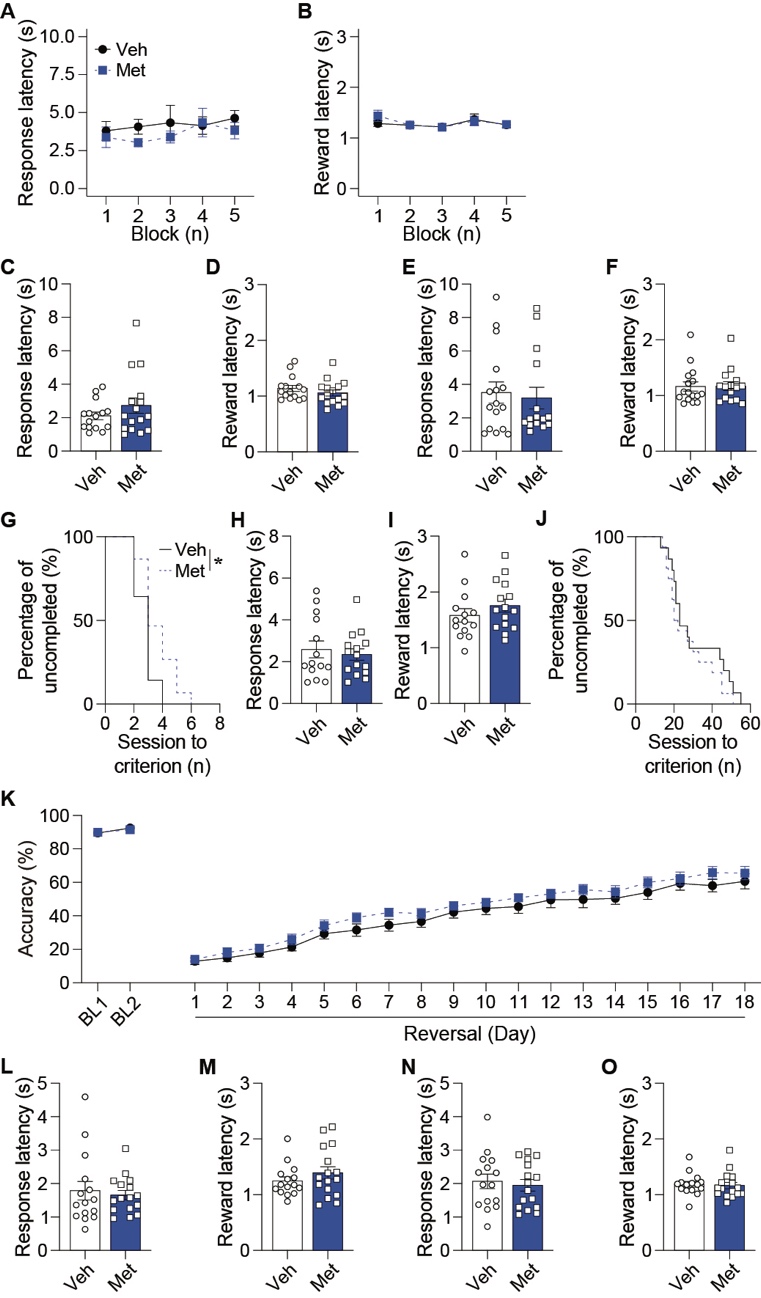
**

**Supplementary Figure 6.** Task performance and motor function of NT mice during the second PAL, VD, and reversal tasks. (A) Response latency (*p* = 0.384) and (B) reward collection latency (*p* = 0.571; mixed effects model) of 22-month-old NT mice (Veh, n = 14; Met, n = 16) in the dPAL task. The block consisted of approximately 300 trials (288–324) as the number of trials per session gradually increased. (C) Response latency (*p* = 0.248), and (D) reward collection latency (*p* = 0.288; t-test) of 24-month-old NT mice (Veh, n = 14; Met, n = 16) in the sPAL task. (E) Response latency (*p* = 0.723) and (F) reward collection latency (*p* = 0.958; t-test) of 25-month-old NT mice (Veh, n = 14; Met, n = 16) in the sPAL retention sessions. (G) Percentage of mice below the criterion of the VD task (*p* = 0.018; log-rant test), (H) response latency (*p* = 0.609), and (I) reward collection latency (*p* = 0.299; t-test) of 25-month-old NT mice (Veh, n = 14; Met, n = 15) in the VD task. (J) Overall accuracy (*p* = 0.185; RM-ANOVA), (K) percentage of mice below the criterion of reversal task (*p* = 0.304; log-rank test), (L) response latency (*p* = 0.669), and (M) reward collection latency (*p* = 0.296; t-test) of 26-month-old NT mice (Veh, n = 15; Met, n = 16) in the reversal task. (N) Response latency (*p* = 0.679) and (O) reward collection latency (*p* = 0.780; t-test) of 29-month-old NT mice (Veh, n = 15; Met, n = 16) in the reversal retention session. Data are presented as mean ± SEM. NT, non-transgenic C57BL/6 mice; Veh, Vehicle; Met, Metformin; BL, baseline.

**
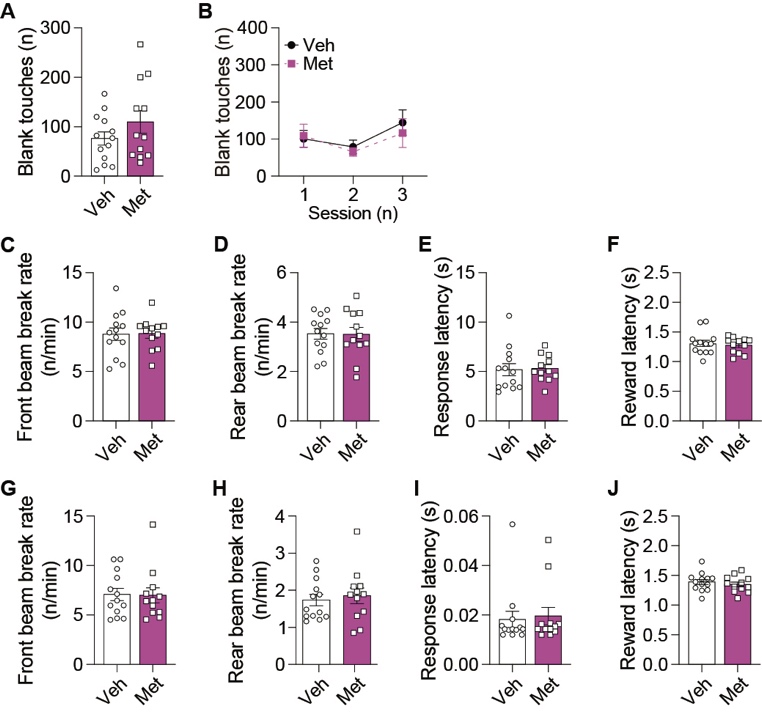
**

**Supplementary Figure 7.** Task performance and motor function of AD mice during the FR and PR schedules. The number of blank touches of (A) 5-month-old AD mice (Veh, n = 13; Met, n = 12) in FR schedule (*p* = 0.213; t-test) and (B) 6-month-old AD mice (Veh, n = 13; Met, n = 12) in PR schedule (*p* = 0.741; RM ANOVA). (C) Front beam break rate (*p* = 0.936), (D) rear beam break rate (*p* = 0.945), (E) response latency (*p* = 0.865), and (F) reward collection latency (*p* = 0.631; t-test) of 5-month-old AD mice in FR schedule. (G) Front beam break rate (*p* = 0.931), (H) rear beam break rate (*p* = 0.651), (I) response latency (*p* = 0.774), and (J) reward collection latency (*p* = 0.481; t-test) of 6-month-old AD mice in PR schedule. AD, 3xTg-AD mice; Veh, Vehicle; Met, Metformin.


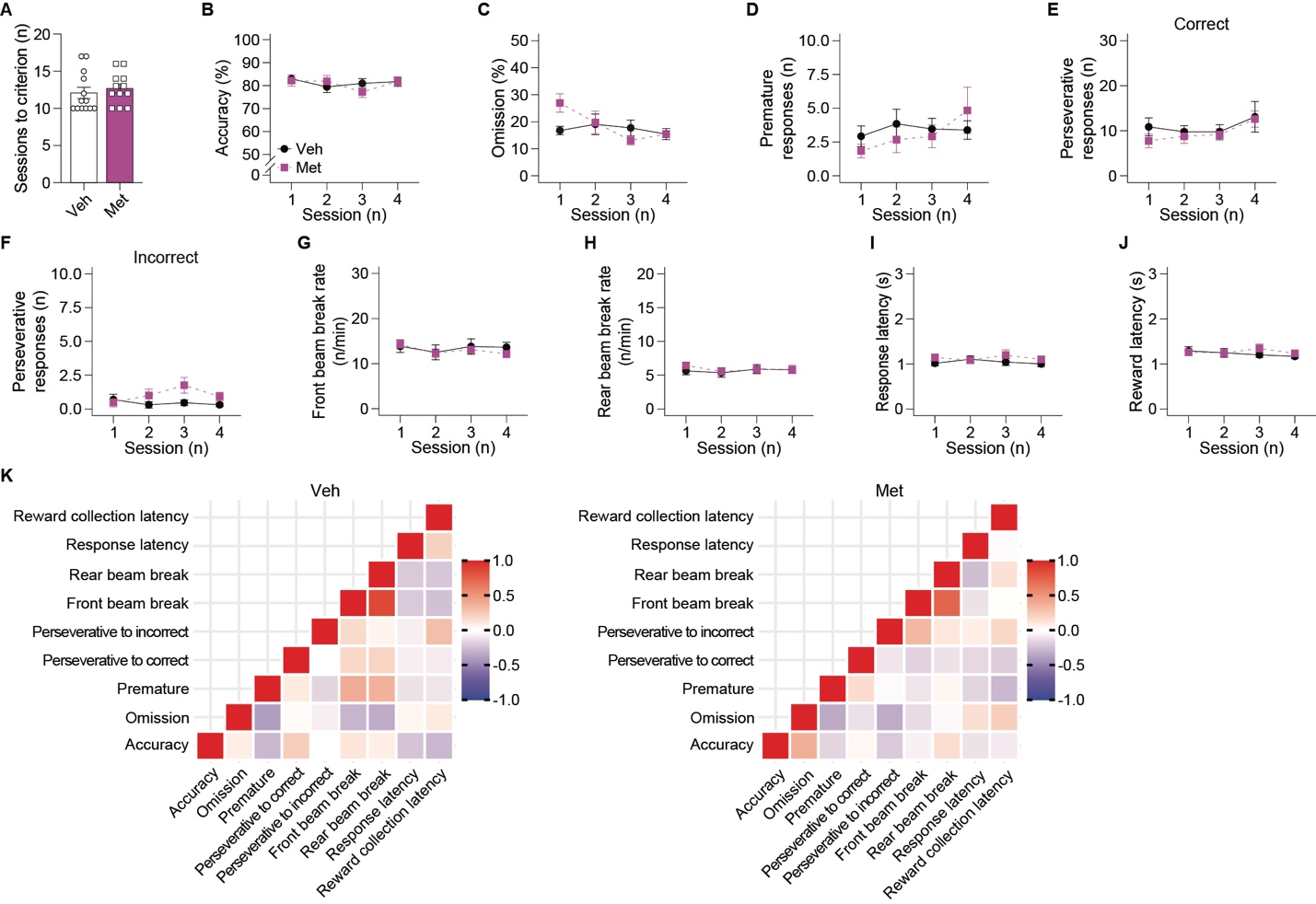


**Supplementary Figure 8.** Task performance and motor function of AD mice during the 5-CSRT task. (A) The number of sessions required to accomplish the criterion of the 5-CSRT task during the acquisition phase of 7-month-old AD mice (Veh, n = 13; Met, n = 12; *p* = 0.533; t-test). (B) Accuracy (*p* = 0.809), (C) omission (*p* = 0.516), and the number of (D) premature responses (*p* = 0.732) and perseverative responses to (E) correct stimuli (*p* = 0.488) and (F) incorrect stimuli (*p* = 0.090; mixed effects model) of 9-month-old AD mice (Veh, n = 13; Met, n = 12) in each session of 5-CSRT probe test. (G) Front beam break rate (*p* = 0.763), (H) rear beam break rate (*p* = 0.752), (I) response latency (*p* = 0.107), and (J) reward collection latency (*p* = 0.560) of 9-month-old AD mice in the 5-CSRT probe test. (K) Heat map representing the relationship between the variables of the 5-CSRT task in Veh (left) and MET (right) groups of 9-month-old AD mice. Data are presented as mean ± SEM. **p* < 0.05 versus Veh. AD, 3xTg-AD mice; Veh, Vehicle; Met, Metformin.

**
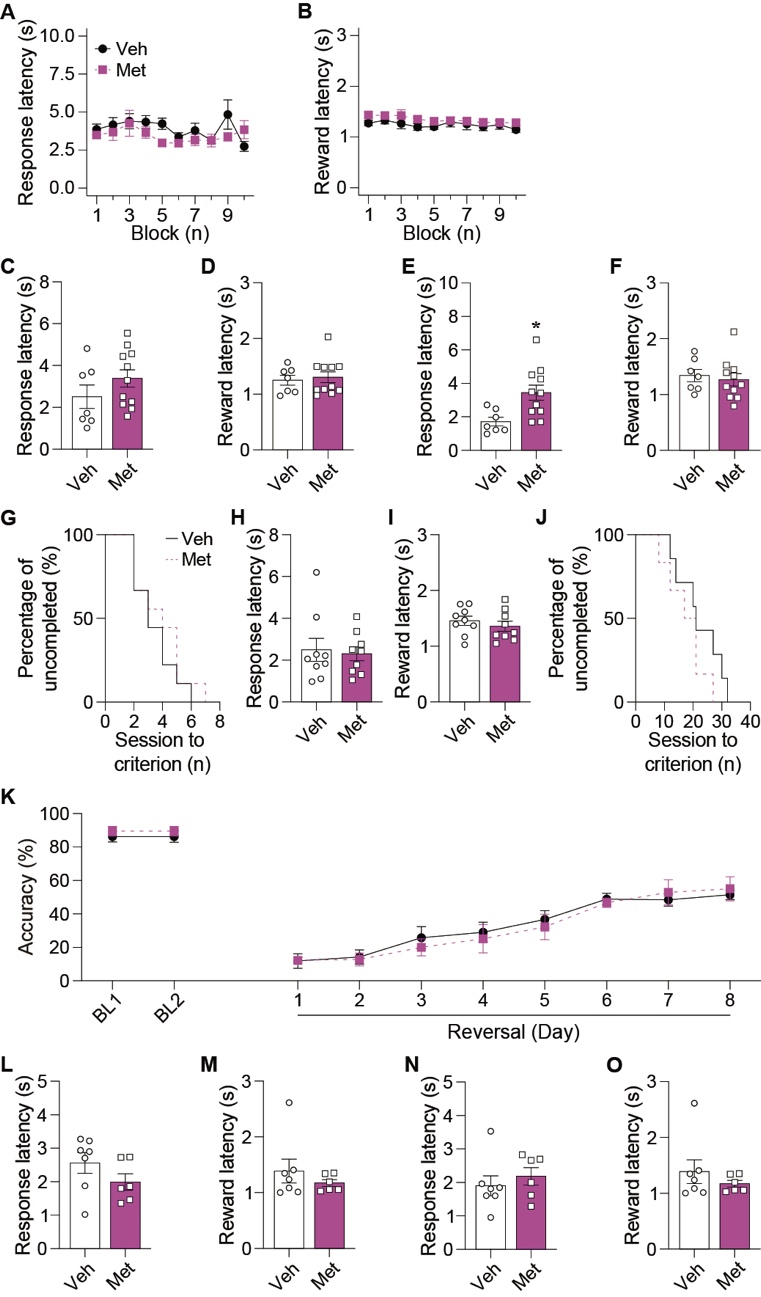
**

**Supplementary Figure 9.** Task performance and motor function of AD mice during the PAL, VD, and reversal tasks. (A) Response latency (*p* = 0.302) and (B) reward collection latency (*p* = 0.135; mixed effects model) of 11-month-old AD mice (Veh, n = 6; Met, n = 10) in the dPAL task. The block consisted of approximately 300 trials (288-324) as the number of trials per session gradually increased. (C) Response latency (*p* = 0.221) and (D) reward collection latency (*p* = 0.712; t-test) of 13-month-old AD mice (Veh, n = 6; MET, n = 10) in the sPAL task. (E) Response latency (*p* = 0.012) and (F) reward collection latency (*p* = 0.653; t-test) of 13-month-old AD mice (Veh, n = 6; Met, n = 10) in the sPAL retention sessions. (G) Percentage of mice below the criterion of the VD task (*p* = 0.428; log-rank test), (H) response latency (*p* = 0.777), and (I) reward collection latency (*p* = 0.439; t-test) of 14-month-old AD mice (Veh, n = 9; Met, n = 9) in the VD task. (J) Overall accuracy (*p* = 0.951; RM-ANOVA), (K) percentage of mice below the criterion of reversal task (*p* = 0.247; log-rank test), (L) response latency (*p* = 0.188), and (M) reward collection latency (*p* = 0.098; t-test) of 14-month-old AD mice (Veh, n = 7; Met, n = 6) in the reversal task. (N) Response latency (*p* = 0.499) and (O) reward collection latency (*p* = 0.386; t-test) of 16-month-old AD mice (Veh, n = 7; Met, n = 6) in the reversal retention session. Data are presented as mean ± SEM. **p* < 0.05 versus Veh. AD, 3xTg-AD mice; Veh, Vehicle; Met, Metformin; BL, baseline.


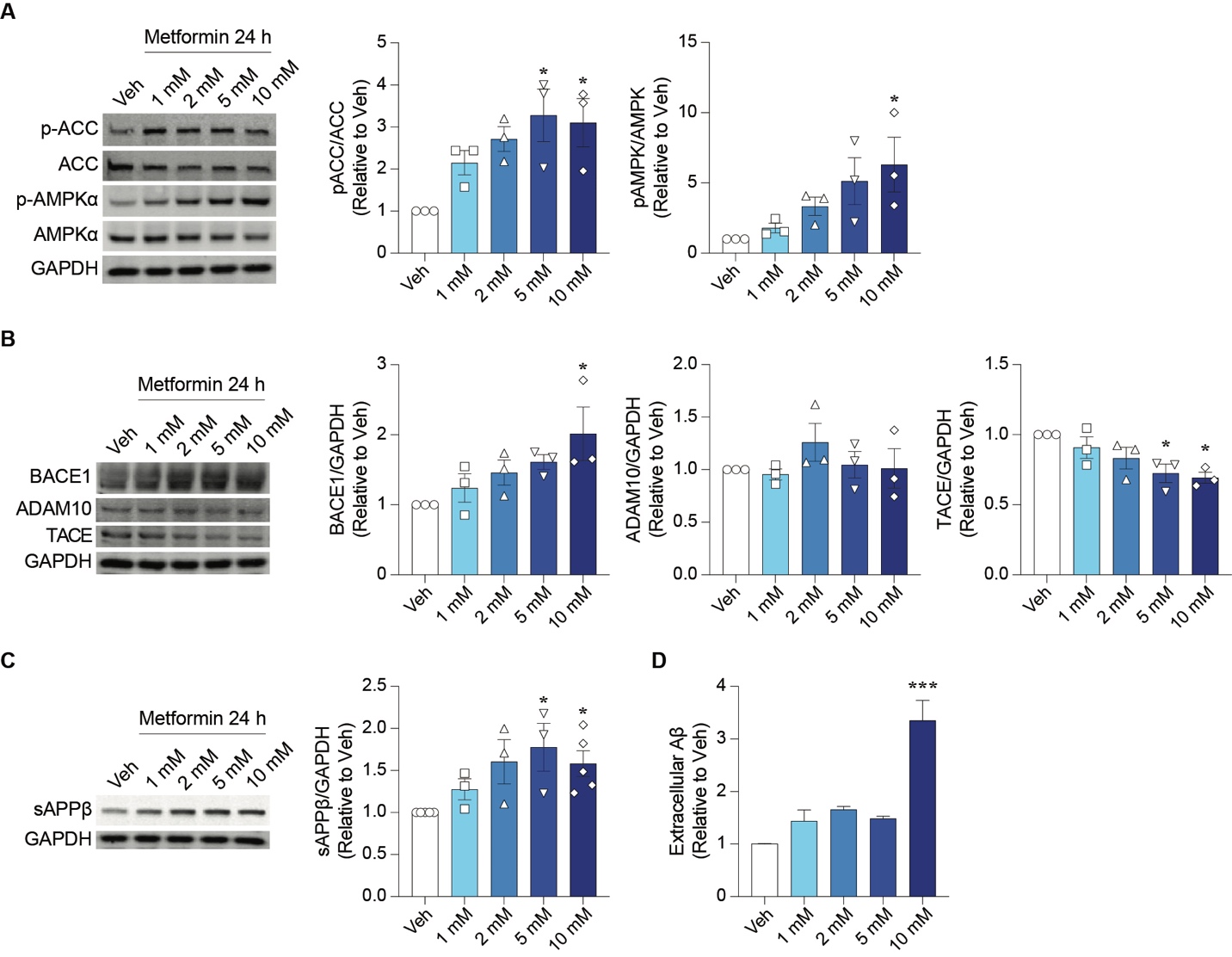


**Supplementary Figure 10.** The effects of metformin on amyloidogenic pathway *in vitro*. Western blot analysis band images and quantification of (A) ACC and AMPKα expression and their phosphorylation levels (3 independent experiment; p-ACC/ACC, *p* = 0.231 in 1 mM, *p* = 0.052 in 2 mM, *p* = 0.011 in 5 mM; *p* = 0.018 in 10 mM; p-AMPKα/AMPKα, *p* = 0.970 in 1 mM, *p* = 0.479 in 2 mM, *p* = 0.103 in 5 mM; *p* = 0.033 in 10 mM) and (B) BACE1, ADAM10, and TACE expression (3 independent experiment; BACE1, *p* = 0.844 in 1 mM, *p* = 0.406 in 2 mM, *p* = 0.201 in 5 mM; *p* = 0.024 in 10 mM; ADAM10, *p* = 0.997 in 1 mM; *p* = 0.469 in 2 mM, *p* = 0.997 in 5 mM; *p* > 0.999 in 10 mM; TACE, *p* = 0.655 in 1 mM; *p* = 0.207 in 2 mM, *p* = 0.026 in 5 mM; *p* = 0.014 in 10 mM; Dunnett’s test). (C) Western blot analysis band images and quantification of sAPPβ in Neuro2a-APP_695_ stable cell line after treatment of metformin for 24 h (3 independent experiment; sAPPβ, *p* = 0.631 in 1 mM, *p* = 0.075 in 2 mM, *p* = 0.019 in 5 mM; *p* = 0.044 in 10 mM; Dunnett’s test). (D) Extracellular Aβ_1-42_ quantification in Neuro2a-APP_695_ after treatment of metformin for 24 h (3 independent experiment; *p* = 0.231 in 1 mM, *p* = 0.052 in 2 mM, *p* = 0.011 in 5 mM; *p* = 0.018 in 10 mM; Dunnett’s test). Data are presented as mean ± SEM. **p* < 0.05, ****p* < 0.001 versus Veh. Veh, vehicle; Met, metformin.

**
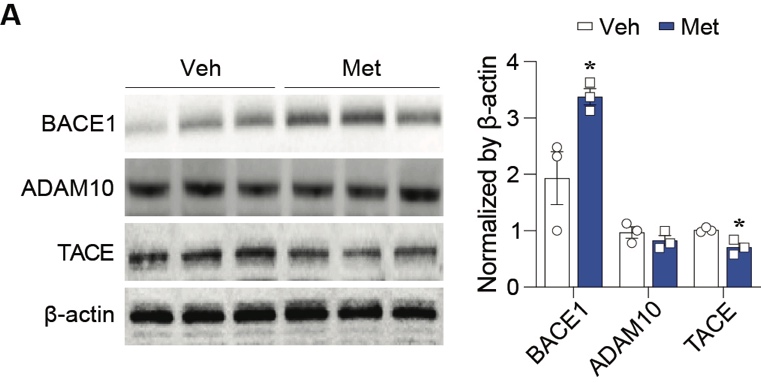
**

**Supplementary Figure 11.** The effects of metformin on amyloidogenic pathway in db/db mice. (A) Western blot analysis band images and quantification of the expression of BACE1, ADAM10, and TACE in male db/db mice (n = 3 per group; BACE1, *p* = 0.042; ADAM10, *p* = 0.344; TACE, *p* = 0.019; t-test) after treatment of 2 mg/ml metformin for 7 days. Data are presented as mean ± SEM. **p* < 0.05 versus Veh. Veh, vehicle; Met, metformin.


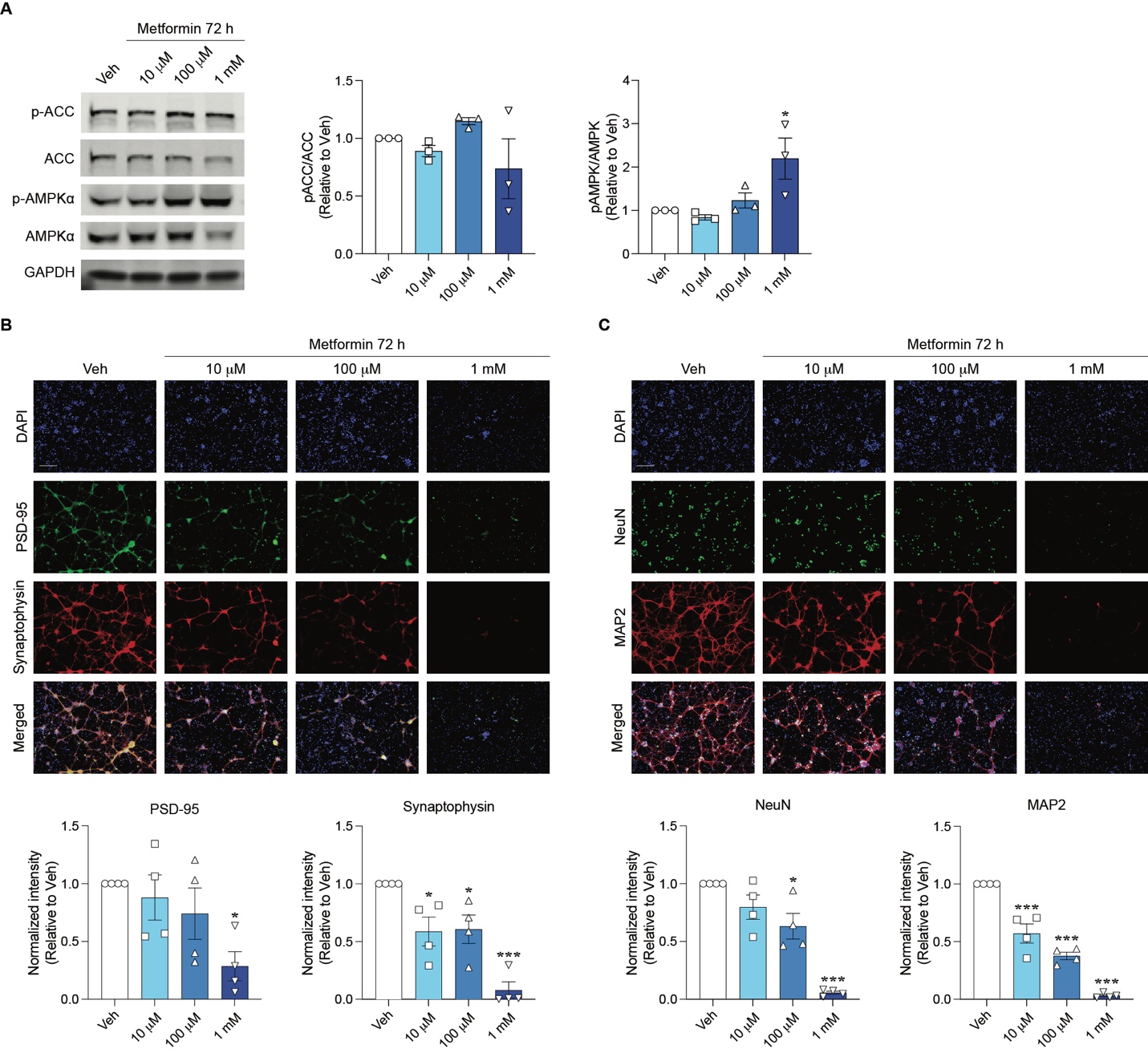


**Supplementary Figure 12.** The effects of metformin on synaptic and neuronal markers in primary neuron culture. (A) Western blot analysis band images and quantification of ACC and AMPKα expression and their phosphorylation levels in mouse primary neuron culture (3 independent experiment; p-ACC/ACC, *p* = 0.887 in 10 μM, *p* = 0.776 in 100 μM, *p* = 0.414 in 1 mM; p-AMPKα/AMPKα, *p* = 0.943 in 10 μM, *p* = 0.859 in 100 μM, *p* = 0.026 in 1 mM; Dunnett’s test). (B) Representative immunocytochemistry images and intensity quantification of PSD-95 (green) and synaptophysin (red) in mouse primary neuron culture (4 independent experiment; PSD-95, *p* = 0.912 in 10 μM, *p* = 0.546 in 100 μM, *p* = 0.022 in 1 mM; synaptophysin, *p* = 0.025 in 10 μM, *p* = 0.032 in 100 μM, *p* < 0.001 in 1 mM; Dunnett’s test). Scale bar: 200 μm. (C) Representative immunocytochemistry images and intensity quantification of NeuN (green) and MAP2 (red) in mouse primary neuron culture (4-6 independent experiment; NeuN, *p* = 0.201 in 10 μM, *p* = 0.014 in 100 μM, *p* < 0.001 in 1 mM; MAP2, p < 0.001 in 10 μM, *p* < 0.001 in 100 μM, *p* < 0.001 in 1 mM; Dunnett’s test). Scale bar: 200 μm. Data are presented as mean ± SEM. **p* < 0.05, ****p* < 0.001 versus Veh. Veh, vehicle.

**Supplementary Table**

**Supplementary Table 1.** The number of mice used in each experiment

| **NT mice with food restriction** | |  |  |
| --- | --- | --- | --- |
| **Experiment** | **Age** | **Veh** | **Met** |
| Weight | Throughout the ages | n = 16 | n = 16 |
| Water (Cage) | 3-month-old | n = 4 | n = 4 |
| Blood glucose concentration | 4- and 29-month-old | n = 16 | n = 16 |
| Survival | Throughout the ages | n = 16 | n = 16 |
| 1^st^ FR task | 4-month-old | n = 16 | n = 16 |
| 1^st^ PR task | 4-month-old | n = 16 | n = 16 |
| 1^st^ 5-CSRT task | 6-month-old | n = 16 | n = 16 |
| 1^st^ dPAL task | 12-month-old | n = 16 | n = 13 |
| 1^st^ sPAL task | 16-month-old | n = 16 | n = 13 |
| 1^st^ sPAL retention sessions | 16-month-old | n = 15 | n = 13 |
| 2^nd^ FR task | 20-month-old | n = 15 | n = 16 |
| 2^nd^ PR task | 20-month-old | n = 15 | n = 16 |
| 2^nd^ 5-CSRT task | 22-month-old | n = 15 | n = 16 |
| 2^nd^ dPAL task | 22-month-old | n = 14 | n = 16 |
| 2^nd^ sPAL task | 24-month-old | n = 14 | n = 16 |
| 2^nd^ sPAL retention sessions | 25-month-old | n = 14 | n = 16 |
| VD task | 25-month-old | n = 14 | n = 15 |
| Reversal task | 26-month-old | n = 15 | n = 16 |
| Reversal retention session | 29-month-old | n = 15 | n = 16 |
| **AD mice with food restriction** | |  |  |
| **Experiment** | **Age** | **Veh** | **Met** |
| Weight | Throughout the ages | n = 14 | n = 12 |
| Water (Cage) | 4-month-old | n = 8 | n = 5 |
| Blood glucose concentration | 5- and 16-month-old | n = 14 | n = 12 |
| Survival | Throughout the ages | n = 14 | n = 12 |
| FR task | 5-month-old | n = 13 | n = 12 |
| PR task | 6-month-old | n = 13 | n = 12 |
| 5-CSRT task | 7-month-old | n = 13 | n = 12 |
| dPAL task | 11-month-old | n = 6 | n = 10 |
| sPAL task | 13-month-old | n = 6 | n = 10 |
| sPAL retention sessions | 13-month-old | n = 6 | n = 10 |
| VD task | 14-month-old | n = 9 | n = 9 |
| Reversal task | 14-month-old | n = 7 | n = 6 |
| Reversal retention session | 16-month-old | n = 7 | n = 6 |
| **AD mice without food restriction** | |  |  |
| **Experiment** | **Age** | **Veh** | **Met** |
| Weight | Throughout the ages | n = 6 | n = 5 |
| Water (Cage) | 4-month-old | n = 2 | n = 2 |
| Blood glucose concentration | 5- and 16-month-old | n = 6 | n = 5 |
| Survival | Throughout the ages | n = 6 | n = 6 |

Veh, Vehicle; Met, Metformin; NT, non-transgenic C57BL/6; AD, 3xTg-AD.

**Supplementary Table 2.** List of antibodies used in this study

| **Primary antibody** | **Species** | **Catalog No.** | **Specificity** | **Supplier** | **Dilution** | **Exp** |
| --- | --- | --- | --- | --- | --- | --- |
| Anti-phospho-AMPKα (Thr172) | Rabbit | 2535 | Monoclonal IgG | Cell Signaling Technology (Danvers, MA, USA) | 1:1000 | WB |
| Anti-AMPKα | Rabbit | 5831 | Monoclonal IgG | Cell Signaling Technology | 1:1000 | WB |
| Anti-phospho-ACC (Ser79) | Rabbit | 11818 | Monoclonal IgG | Cell Signaling Technology | 1:1000 | WB |
| Anti-AMPKα1 | Rabbit | 2795 | Polyclonal  IgG | Cell Signaling Technology | 1:1000 | WB |
| Anti-AMPKα2 | Rabbit | 2757 | Polyclonal  IgG | Cell Signaling Technology | 1:1000 | WB |
| Anti-BACE1 | Rabbit | 5606 | Polyclonal  IgG | Cell Signaling Technology | 1:1000 | WB |
| Anti-pGSK3β (Ser9) | Rabbit | 9322 | Polyclonal  IgG | Cell Signaling Technology | 1:1000 | WB |
| Anti-GSK3α/β | Rabbit | 5676 | Monoclonal IgG | Cell Signaling Technology | 1:1000 | WB |
| Anti-Aβ (D54D2) | Rabbit | 8243 | Monoclonal IgG | Cell Signaling Technology | 1:100 | IHC |
| Anti-phospho-tau (Ser396) | Mouse | 9632 | Monoclonal IgG | Cell Signaling Technology | 1:1000 | WB |
| Anti-tau46 | Mouse | 4019 | Monoclonal IgG | Cell Signaling Technology | 1:2000 | WB |
| Anti-phospho-tau (Ser199/202) | Rabbit | 44-768G | Polyclonal  IgG | Invitrogen (Carlsbad, CA, USA) | 1:1000 | WB |
| Anti-phospho-tau (Thr231) | Rabbit | 44-746G | Polyclonal  IgG | Invitrogen | 1:1000 | WB |
| Anti-ACC | Mouse | 05-1098 | Monoclonal IgG | Merck Millipore (Burlington, MA, USA) | 1:1000 | WB |
| Anti-Aβ (H-43) | Rabbit | sc-9129 | Polyclonal  IgG | Santa Cruz Biotechnology, Inc. (Dallas, TX, USA) | 1:1000 | WB |
| Anti-ADAM10 | Mouse | sc-28358 | Monoclonal IgG | Santa Cruz Biotechnology, Inc. | 1:1000 | WB |
| Anti-TACE | Rabbit | sc-13973 | Polyclonal  IgG | Santa Cruz Biotechnology, Inc. | 1:1000 | WB |
| Anti-phospho-tau (Ser262) | Rabbit | sc-32828 | Polyclonal  IgG | Santa Cruz Biotechnology, Inc. | 1:1000 | WB |
| Anti-phospho-tau (Ser356) | Rabbit | sc-101814 | Polyclonal  IgG | Santa Cruz Biotechnology, Inc. | 1:1000 | WB |
| Anti-β-actin | Mouse | sc-47778 | Monoclonal IgG | Santa Cruz Biotechnology, Inc. | 1:5000 | WB |
| sAPPβ | Rabbit | SIG-39138 | Polyclonal  IgG | Biolegend | 1:1000 | WB |
| Anti-APP or Aβ (6E10) | Mouse | SIG-39320 | Monoclonal IgG | Biolegend (San Diego, CA, USA) | 1:1000 | WB |
| Anti-GAPDH | Mouse | SIG-166574 HRP | Monoclonal IgG | Santa Cruz Biotechnology, Inc. | 1:1000 | WB |
| Anti-PSD-95 | Mouse | 124 011 | Monoclonal IgG | Synaptic Systems (Göttingen, Germany) | 1:500 | ICC |
| Anti-Synaptophysin | Rabbit | ab32127 | Monoclonal IgG | Abcam (Cambridge, UK) | 1:500 | ICC |
| Anti-NeuN | Rabbit | PA5-78499 | Polyclonal  IgG | Invitrogen | 1:500 | ICC |
| Anti-MAP2 | Guinea pig | 188 004 | Monoclonal IgG | Synaptic Systems | 1:500 | ICC |
| **Secondary antibody** | **Conjugate** | **Catalog No.** | **Specificity** | **Supplier** | **Dilution** | **Exp** |
| Goat-anti-rabbit | HRP | 7074 | IgG | Cell Signaling Technology | 1:5000  or 1:10000 | WB |
| Horse-anti-mouse | HRP | 7076 | IgG | Cell Signaling Technology | 1:5000  or 1:10000 | WB |
| Goat-anti-rabbit | Alexa Fluor 488 | 111-546-144 | Polyclonal  IgG | Jackson Immunoresearch Lab, Inc. (West Grove, PA, USA) | 1:100 | IHC |
| Donkey-anti-rabbit | Alexa Fluor 488 | 711-546-152 | Polyclonal  IgG | Jackson Immunoresearch Lab, Inc. | 1:500 | ICC |
| Donkey-anti-rabbit | Alexa Fluor 594 | 711-586-152 | Polyclonal  IgG | Jackson Immunoresearch Lab, Inc. | 1:500 | ICC |
| Donkey-anti-mouse | Alexa Fluor 488 | 715-546-150 | Polyclonal  IgG | Jackson Immunoresearch Lab, Inc. | 1:500 | ICC |
| Donkey-anti-guinea pig | Alexa Fluor 594 | 706-586-148 | Polyclonal  IgG | Jackson Immunoresearch Lab, Inc. | 1:500 | ICC |

Exp, Experiment; WB, Western blot; IHC, Immunohistochemistry; ICC, Immunocytochemistry.

**Supplementary Table 3.** List of metabolites assessed via multiple linear regression analysis

| **Abbreviation** | **Compound** |
| --- | --- |
| PALC | Palmitoylcarnitine |
| CAR | L-Carnitine |
| TRG | TRIGONELLINE |
| GUA | Guanine |
| KYN | Kynurenine |
| Glu Gln | Gamma-Glutamylglutamine |
| MTF | Metformin |
| Deoxy-CAR | DEOXYCARNITINE |
| THHCA | (1S,3R,4S,5R)-4-{[(2E)-3-(3,4-dihydroxyphenyl)prop-2-enoyl]oxy}-1,3,5-trihydroxycyclohexane-1-carboxylic acid |
| THPCX | N-((octahydro-1H-quinolizin-1-yl)methyl)-2,4,5,6-tetrahydrocyclopenta[c]pyrazole-3-carboxamide |
| PIP | L-Pipecolic acid |
| PRPT | Prespatane |
| Lys | Lysine |
| DPA | Diphenylamine |
| Ado | Adenosine |
| 5-AVAB | 5-Aminovaleric acid betaine |
| GMP | Guanosine 5'-monophosphate |
| Glu | L-Glutamic acid |
| NAP | N-Acetylphenylalanine |
| DEP | Diethyl phthalate |
| DBP | Dibutyl phthalate |
| Met | Methionine |
| SLCH | Sulochrin |
| Guo | Guanosine |
| Arg | Arginine |
| PA | Pantothenic acid |
| Glycero-PC | sn-Glycero-3-phosphocholine |
| MMCO | 6-methoxy-4-methyl-2H-chromen-2-one |
| INO | Inosine |
| CA | Cholic Acid |
| CIT | Citric acid |
| AH | Hippuric acid |
| CDCA | Chenodeoxycholic acid |
| HDCA | Hyodeoxycholic acid |
| GSH | Glutathione |
| Jas | Jasmonic acid |
| TCA | trans-Cinnamaldehyde |
